# Six decades of longitudinal health knowledge production: a systematic review on Nordic birth cohort studies

**DOI:** 10.1080/22423982.2023.2278815

**Published:** 2023-11-27

**Authors:** Anna Reetta Rönkä, Annukka Sailo, Noora Hirvonen

**Affiliations:** a Faculty of Education and Psychology and History of Sciences and Ideas, Faculty of Humanities, University of Oulu, Oulu, Finland; b History of Sciences and Ideas, Faculty of Humanities, University of Oulu, Oulu, Finland; c Information Studies, Faculty of Humanities, University of Oulu, Oulu, Finland

**Keywords:** Birth cohort, cohort studies, systematic review, knowledge production, longitudinal studies, Nordic countries, Arctic

## Abstract

This systematic review (a) identifies birth cohort studies (BCSs) established in the Nordic countries, (b) describes their basic characteristics, and (c) explores how these characteristics have evolved over time, discussing their implications to knowledge production. To identify Nordic BCSs, cohort databases and relevant scientific articles were systematically searched and screened.

The review shows that since 1959, more than 600,000 index children have participated in the 79 Nordic BCSs (22 Danish, 20 Finnish, 12 Norwegian, 24 Swedish, one Icelandic), over half of them still ongoing. The Nordic BCSs cover a wide geographical area including the Nordic Arctic. The topics of BCSs have varied over time but most have focused on examining the developmental origins of diseases. A quarter of them had a general scope, while the rest started with a specific focus, commonly atopic diseases. All BCSs collected questionnaire and/or interview data and over 60% of the BCSs announced exclusion criteria for participants, typically insufficient language proficiency.

NBCSs have produced crucial scientific knowledge for over six decades, but there are underutilised opportunities including systematic interdisciplinary collaboration, inclusion of children’s own views of their health and well-being, intergenerational data collection, and specific knowledge of Arctic indigenous peoples and other minorities.

## Introduction

Longitudinal birth cohort studies (BCS) provide unique opportunities to produce scientific knowledge on the associations and causal relationships between early-life exposures with later health and wellbeing [1,2] extending all the way to old age [3]. In a birth cohort study, longitudinal data is collected from a group of people born at a certain time in a defined geographical location by active measures such as clinical studies and surveys, and/or passive measures like hospital records and other registers, with follow-ups over a period of time [4]. Recruitment for these studies happens during pregnancy, at birth [5], or later in life. A pregnancy cohort recruits pregnant women and follows them and their children prospectively whereas in a birth cohort study, recruitment happens at birth, and data collection concentrates on the children [6]. The length of the study can vary from months to years or even decades, until the passing of the cohort member, the “index child” [7]. In a third form of birth cohort study the “index child” is recruited later in life and is usually followed-up both retrospectively and prospectively [5]. In this review, we will refer to all these three study types as birth cohort studies (see [5].

BCS as a research design, usually with long-term follow-up with multiple ways of data collection, provides insights into the development of different kinds of exposures for later development of disease, including genetic, socio-economic, environmental, and lifestyle factors [1,8]. This way, BCSs enable the identification of key exposures and effective preventive methods of different types of diseases and sources of ill-being which benefits individuals and societies [9].

The UK is the pioneering country in establishing birth cohort studies [10], implementing the first large birth cohort study, The National Birth Cohort (MRC National Survey of Health and Development) in 1946, soon after World War II. Outside of the UK, the efforts of conducting birth cohort studies were sporadic and rare [11], but a dramatic rise in BCS was witnessed in the 1990s [4], especially in Northern Europe and in other high-income countries such as the US [12]. This rise has happened in parallel with increased interest in the early-life developmental origins of diseases [1]. From the turn of the millennium, genetic and epigenetic variables have been increasingly included in the studies (see [13], and many birth cohorts have created or have collaborated with biobanks to collect and store biological specimens to better investigate the interplay between genetic, lifestyle, and environmental factors in wellbeing and health [14,15].

The Nordic countries, Denmark, Finland, Norway, Sweden, and Iceland, have long traditions of conducting BCSs [5], some of them being among the oldest and largest in the world [1,3]. Considering the exceptional prominence of BCSs in this geographical region and their scientific and health policy impact [16], it is timely to conduct a systematic review of them. In this review, we will present central characteristics of Nordic BCSs and examine how these characteristics have evolved over time and pay attention to the scientific knowledge production about and with BCS research participants.

Previous systematic reviews of birth cohort studies have focused on specific countries [15,17,18] or larger regions such as the Gulf Cooperation Council countries [19]; Sub-Saharan Africa [4] and Europe [1,5,6,20] or have had a specific focus, such as environmental exposures [21] or asthma and atopic diseases [22].

Some of the Nordic cohorts are included in these previous review studies (e.g [5,22], but they are not systematically covered. Moreover, the Nordic countries include Arctic regions with indigenous populations of Sámi and Greenlandic Inuits, whose participation has not been discussed in previous BCS reviews.^1^^1^Weihe et al. [73] provided an overview of ongoing cohort and dietary studies in the Arctic, but while describing some birth cohorts, some birth cohorts located in the Arctic areas in the Nordic countries were missing.

### Nordic welfare states as research setting

The Nordic countries are technologically developed, politically stable democracies with gross domestic products above the European average [23] and share a lot of common history and culture. All five implement a version of the Nordic welfare model, which is characterised by universalism, high level of taxation and welfare expenditures, high employment rate, active labour market policies, and a high degree of equality [24,25]. These countries provide free education and universal and predominantly publicly financed health-care services.

The Nordic countries are particularly well suited for conducting birth cohort studies. As welfare societies, they share a strong interest in monitoring and improving the health and well-being of their population, and they have invested substantially in research and research infrastructures. The high legitimacy of and sense of trust in the state and its health institutions [26] have likely contributed to high participation rates in birth cohort studies in the Nordic countries (e.g [27]. But even more importantly, the data-gathering processes are greatly enhanced by the long traditions of maintaining nationwide registers and establishing personal identification numbers for citizens and permanent residents. This makes it possible to link data from several registers and other sources with register-based information at an individual level [28,29]. In addition, standardised maternity care practices produce large amounts of widely comparable medical data on every pregnancy and infant, and facilitate the recruitment of mothers and babies in a birth cohort study (e.g [30].

While the Nordic welfare states perform well in terms of life expectancy and overall population health, they also experience significant health inequalities. Besides inequalities related to socioeconomic differences, especially in Sweden and Finland, there are health disparities between southern and northern parts of the country and between urban and scattered settlement areas [31]. Northern and Arctic areas of Nordic countries face specific conditions and challenges affecting population health and wellbeing, such as scarcely populated areas, long distances, strong female outmigration and decreasing population rates, higher unemployment [32,33], cold climate, high number of substance abuse and interpersonal violence, as well as suicides [34,35].

In the Nordic Arctic, two indigenous peoples are residing: Greenlandic Inuits in semi-autonomous area of Greenland, and Sámi people. According to two global reviews, indigenous people´s health and wellbeing are poorer in comparison to majority populations living in the same countries [35,36]. Especially in Greenland, its Inuit population has more health problems and lower life expectancy than the rest of the Nordic region [37]. With the Sámi in the Nordic countries,^2^^2^The traditional settlement area of Sámi people, Sápmi, stretches over the northern parts of Norway, Sweden, and Finland, and a small portion covers the Kola Peninsula in Russia, but a large part of the Sámi lives outside of this area [79]. only minor health differences compared to non-Sámi populations have been found [38]. Yet, there are special challenges and threats to Sámi people and their health, connected to, for instance, the cumulative effects of resource exploitation, climate change, and conflicts with the majority society [38].

### Aim and objectives

Given the unique context of the Nordic countries, their long tradition of establishing birth cohorts, and the fact that they host some of the world’s largest birth cohorts, it is surprising that to date, no systematic reviews of Nordic birth cohort studies have been conducted. Reviews of birth cohort studies are important as they allow us to recognise research trends and knowledge gaps and help in the identification of the strengths and weaknesses of the studies [20,39]. Moreover, they offer information for future cross-cohort collaboration, which is desirable as combined analysis of data may bring efficacy to research efforts, improve statistical power and reduce publication bias as well as improve contrast and diversity in exposure and outcome measures [1,20,21].

The aim of this systematic review is to identify and review birth cohort studies conducted within the five Nordic countries, Finland, Sweden, Norway, Iceland, and Denmark, including the autonomous areas of Faroese Islands and Greenland (Denmark) and Åland Islands (Finland). The objectives are to (a) identify existing birth cohort studies, (b) describe their basic characteristics including geographical locations, study focus, types of data collected, and exclusion criteria for study participation, and to (c) explore how these characteristics have evolved over time. These issues are discussed in terms of their potential implications for the scientific knowledge produced with the birth cohort data, especially considering research participation; the review takes into consideration the regional coverage, eligibility criteria for the participants and the respondents to the questionnaires and interviews. As such, we examine the participation in the knowledge production process in BCS, an aspect often neglected in birth cohort reviews.

Even though some of the Nordic birth cohort studies are included in earlier reviews, a lot of information about them is missing. We seek to identify Nordic pregnancy and birth cohort studies that have not been included in previous reviews and to reach a larger variety and representativeness, we also include BCSs where recruitment happened later in life. Moreover, we do not restrict the initiation date of BCSs, allowing the inclusion of the older birth cohorts.

## Materials and methods

To ensure transparent reporting, the PRISMA guidelines (Preferred Reporting Items for Systematic reviews and Meta-Analyses for Protocols) for systematic reviews were followed [40] Detailed PRISMA checklists are found in Appendix 4 and Appendix 5.

### Data sources and study selection

To identify relevant original research articles, EBSCO, Scopus, and ProQuest citation databases were systematically searched 26^th^–27^th^ May 2020 using a strategy where the search term “birth cohort” was combined with terms describing the Nordic countries (Finland, Finnish, Sweden, Swedish, Norway, Norwegian, Denmark, Danish, Iceland, Icelandic). No language or publication status restrictions were imposed at this stage, but the search was directed to titles, abstracts, and keywords and restricted to peer-reviewed research articles. The search was updated with a similar search strategy on the 16^th^ of May 2022.

To be included in the review, the articles needed to meet the following criteria: they had to a) be peer-reviewed and published in English, Finnish, Swedish, Norwegian, Icelandic or Danish; b) report empirical research on a birth cohort study with a study population consisting of a group of people born in specified time and place; including c) two or more types of data, for example, surveys/interviews, clinical data, biological specimen collection, or data from psychological tests; d) more than two follow-ups over a one year period, and e) a sample of at least 200 participants.

An article was excluded if it a) was not an original empirical research article, b) did not report a birth cohort study (was e.g. a case-control study, randomised clinical trial), reported a BCS including c) only passive types of data (registers, hospital or other types of records, national statistical data), d) only one type of data (e.g. only survey data), or e) participants outside the Nordic countries or more than one country as, for the purposes of this review, we wanted to centre on individual Nordic cohorts only.

**Figure 1.**
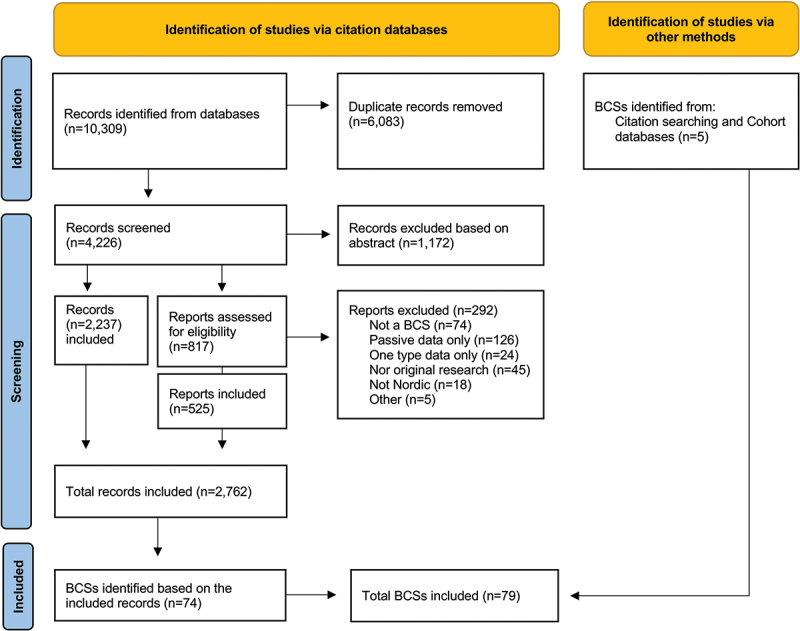
Flow chart of the phases of the literature search and selection^3^

The search resulted in altogether 10,309 literature references (8,901 from the original and 1,408 from the update search), and after the removal of duplicates 4,226 unique articles were identified (3,562 from the original, and 664 from the update search, see Figure 1). The publications were used to detect birth cohort studies and study programmes meeting the predetermined inclusion criteria. In other words, the aim was to identify individual birth cohort studies from the published empirical articles, rather than examining the publications as such.

Information based on the abstracts (*n* = 4,226) of studies included in the review were inspected independently by the authors (Authors 1 and 3 after the first search, and Authors 1, 2, and 3 after the update search). If the abstract did not yield sufficient information on the birth cohort study or the reviewer was unsure about its inclusion, the full article was retrieved and read. Based on abstract screening, 1,172 articles were excluded and 2,237 included. Moreover, 817 full papers were reviewed and 292 of them were excluded. In total, 2,762 articles were included for further analysis. Based on this screening, 74 BCSs were identified.^3^^3^
Source: Page MJ, McKenzie JE, Bossuyt PM, Boutron I, Hoffmann TC, Mulrow CD, et al. The PRISMA 2020 statement: an updated guideline for reporting systematic reviews. BMJ 2021;372:n71. doi: 10.1136/bmj.n71.

Moreover, web-based databases [41,42] which have previously mapped birth cohorts globally were explored, following the search protocol of Larsen et al. [1]. In addition, potentially relevant birth cohort studies were identified from other sources through citation chaining, that is, seeking relevant birth cohorts from the retrieved publications. This resulted in the identification of five additional BCSs.

Descriptive information of the total of 79 BCSs was gathered from individual articles, cohort webpages, and cohort profile papers, commonly published in *International Journal of Epidemiology*, and cross-checked by all authors. In all phases of study selection, a consensus was reached in discussions among all authors.

### Data extraction

Once a birth cohort study had been included, information was extracted from the articles and other sources using a data extraction sheet outlined for the purposes of this review. Guided by the objectives of the review, the following information was sought from each birth cohort study (see Appendix 1): Country; BCS name; Timing; Region of the BCS; Number of index children; Initial topic; Whether the topic of the cohort study was general or specific; Age of participants at recruitment (pregnancy, at birth, later in life/years); Age at follow-ups; Types of data; Respondents on questionnaires (including surveys and diaries) or interviews; and Exclusion criteria or specific inclusion criteria.

## Results

In total, 79 birth cohort studies met our inclusion criteria. Of the cohorts, 20 were Finnish (F1–F20^4^^4^Each cohort was assigned a code with a letter referring to the country and a running number in ascending order.), 12 Norwegian (N1–N12), 24 Swedish (S1–S24), 22 Danish (D1–D22), of which one from Faroese Island and two from Greenland, and one Icelandic (I1). Eight of the cohorts were geographically located in the Arctic areas. In addition, there were altogether seven national cohorts which included also Arctic areas. All the included BCSs are listed in Appendix 1, which depicts their central characteristics.

### Study designs and topics

The majority of the birth cohort studies included in the review were prospective mother/parent-child BCSs. In them, a parent, commonly the mother, and more rarely mother together with the father/partner, was recruited during pregnancy (n = 30) or soon after the birth of their child (n = 26). The data collection started during pregnancy with the mother or upon the birth of the index child with both mother and the child and continued in follow-ups. In 29% (n = 23) of the birth cohort studies recruitment happened years or decades after the birth of the index child. Most of these studies looked also back in time and included retrospective data collection.

Nordic birth cohort studies have predominantly had a medical focus. According to their initial aims, a third (*n* = 16) of the reviewed mother/parent-child BCSs had a general developmental origins of health and disease, “DOHaD”, (see [13; 15]) approach, covering a broad range of prenatal and/or early life exposures on later child development, health, and wellbeing, while the rest (*n* = 40) centred on more specific exposures and/or outcomes (see Table 1 and Appendix 2). Outcome-specific studies focused on the risk factors for and the development of specific diseases, 13 of them on atopic diseases. Exposure-specific studies concentrated on the impact of maternal medical conditions, lifestyles, and pollutants, for example, on the later development of the index child. Moreover, six BCSs specified on mental health including psychiatric problems of the index child or the mother. Of the BCSs commenced later in life, four had general focus on the ageing process or health at an older age while 19 had more specific focus. Of the specific BCSs, six focused on cardiovascular diseases and eight on mental health or behavioural/social matters including the effects of inequalities and risk factors for later mental and behavioural problems.

**Table 1.** Characteristics of 79 Nordic birth cohort studies divided by age of enrolment.

		Number of cohort studies by age of enrollment	
Characteristics		Pregnancy (n=30) or at birth (n=26), N=56	Later in life (> 1 years), N=23
Number of index children	200-499	8	1
	500-999	8	3
	1000-1999	9	7
	2000-4999	14	8
	5000-9999	9	1
	10 000-99 999	5	3
	100 000-	3	-
Types of data collected	Biological	42	16
	Clinical/physical examination	33	18
	Questionnaire	54	22
	Interview	18	14
	Psychological/psychiatric/neurological/cognitive test/evaluation	14	14
	Registers	36	15
	Environmental	8	-
Respondents on questionnaires/interviews	Mother	56	7
	Father	37	5
	Index child	19	22
	Healthcare professional	17	8
	Teacher	5	4
Exclusion/specific inclusion criteria	Non-sufficient language proficiency (excl)	22	4
	Non-sufficient health (excl)	20	1
	At risk (incl)	11	3
	Sex, Ethnicity, Age (excl)	7	5
Initial topic	General	16	4
	Specific	40	19
	*Atopic diseases*	13	1
	*Mental/social/behavioral*	6	8
	*Cardiovascular diseases*	-	6
	*Nutrition/diet*	4	-
	*Diabetes I and associated immune-mediated diseases*	3	-
	*Microbiome*	3	-
	*Pre-term birth*	2	2
	*Pollutants*	2	-
	*Other (oral health, influenza A, children conceived by assisted reproductive technology, sleep)*	7	2

It should be noted that many of the specific birth cohort studies extended their initial focus to cover other subjects. For example, The GLAKU birth cohort (F11) was initially designed to study maternal intake of glycyrrhizin in liquorice, as glycyrrhizin is an inhibitor of cortisol metabolism and might be related in the aetiology of low birth weight [43] but, later, the collected data has been utilised for studying a variety of topics including early life origins of mental health [44].

The typical study designs, characteristics, and topics of the BCSs varied to some extent both between the countries and over time. The recruitment of older children or adults as index children was common in the first two decades of Nordic birth cohort studies. However, the very first Nordic birth cohort study, the Danish Copenhagen Perinatal Birth Cohort (D1), initiated in 1959, was a prospective study with general DOHaD aims and recruited mothers during pregnancy (see Appendix 2).^5^^5^
The rows represent individual birth cohorts organised by country, the columns represent years. Danish cohorts are coloured in orange, Finnish in blue, Norwegian in green, Swedish in yellow and one Icelandic cohort in grey. Lighter shades indicate recruitment during pregnancy or at birth, darker shades indicate recruitment later in life.

**Figure 2.**
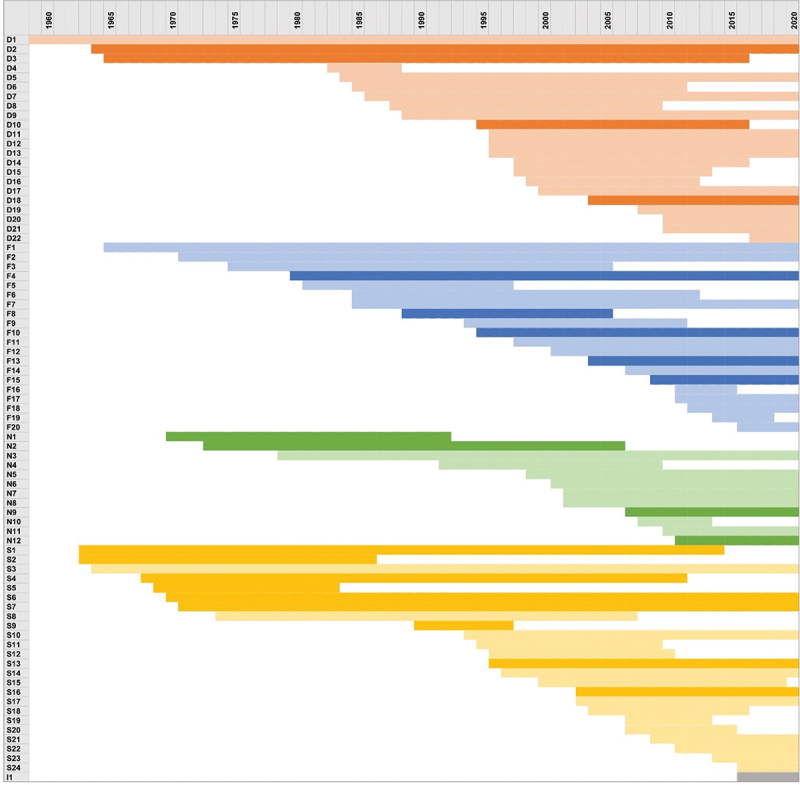
Timeline of the Nordic birth cohorts.^5^

The 1980s seems to have started the golden age of birth cohort studies in Denmark (six new studies) and Finland (five new studies). The 1990s was a prolific decade in the other Nordic countries as well (Figure 2), as 18 birth cohort studies were established, comprising over 290,000 new index children. In the first decade of the 2000s, as many as 19 new birth cohorts were established but with approximately only 55,000 new index children. During these decades, the topics and study designs diversified. However, the 1990s and the early 2000s were especially marked by the number of new cohort studies on atopic diseases – altogether over 10 new studies were launched (e.g. D14, D15, D19, S10, S12, S13, S15, S18, N4). During the same decades, mental health and social issues (S11, D17, D18, N9, N10, S21) and the general health of elderly people (D10, F10, S16) were popular topics. Moreover, studies established in that era addressed the effects of diet on specific diseases (F9) and the effects of specific foods on general child development (F11) as well as gut microbiota development (N8). The effects of preterm birth (F13, F15) and pollutants (N7, S4) were also studied. Some studies focused on the risk factors of specific conditions such as diabetes and other immune-mediated diseases (N6, S14, S15), and cardiovascular diseases (S9).

In the 2010s, only three large scale general prospective birth cohort studies were initiated (D20, F18, S24). Otherwise, studies focused on specific topics including sleep (F16) and postpartum mental health (N10), the effects of stress on harmful behavioural outcomes among adolescents (I1), brain development (F17), epigenetic alterations in children conceived with assisted reproductive technology (S22), the effects on maternal H1N1 infection (N11), and development of allergy/asthma (S23).

Finland and Denmark have had many longitudinal birth cohort programmes with general aims and multiple follow-ups. Sweden and Norway, in contrast, have had fewer programmes with general aims. The high number of allergy and/or asthma cohorts in Sweden is particularly notable. Norway differs from Sweden, Finland, and Denmark in having fewer birth cohorts and a proportional emphasis on examining the effects of pollutants (N4, N7). The only Icelandic birth cohort (I1), a study with 15/16-year-olds focusing on the influence of stress on harmful behaviour, was established as late as 2016. In Finland and Sweden, the latest birth cohort studies were established in 2016 (F20, S24), in Denmark in 2017 (D21), and in Norway in 2011 (N12). In recent years, no new birth cohort studies have been initiated in the Nordic countries.

### Sample sizes and geographical distribution of the cohorts

**Figure 3.**
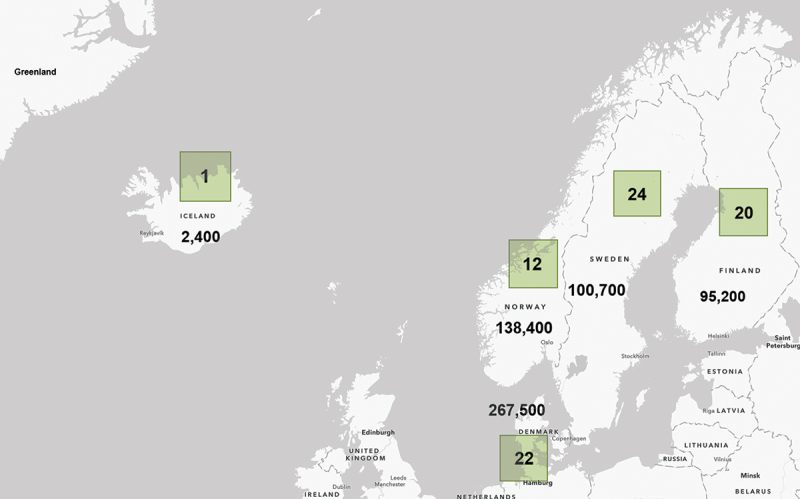
The number of birth cohort studies by country (in green rectangles) and approximate total number of index children recruited in them.

The sample sizes between the BCSs varied considerably, ranging from 205 (F5) to 114,000 (N5). Sweden has established the largest number of birth cohort studies (*n* = 24) among the Nordic countries, but it has enrolled fewer participants (approximately 100,000) in its cohorts than Denmark and Norway (see Figure 3). In Denmark alone, over 260,000 index children have been recruited, and on top of them, mothers and fathers. Three Nordic cohorts (Aarhus Birth Cohort, Denmark; Danish National Birth Cohort DNBC, Denmark; and Norwegian Mother, Father and Child Cohort Study MoBa, Norway) have had 100,000 or more participants each. Birth cohort studies are established in specified geographical locations, ranging from hospital districts and cities to counties, larger regions, and whole countries. Only seven of the reviewed studies were nationwide, among them the DNBC and MoBa. MoBa is one of the most extensive Nordic BCS with 12 follow-ups since its initiation.

Since the acreage of the Nordic countries is relatively large, and the average population density very low (ranging from 0,24 inhabitants/km2 in Greenland and 3,33 inhabitants/km2 in Iceland to 137 inhabitants/km2 in Denmark), it is not surprising that different regions have not been equally represented in the Nordic birth cohort studies. In Finland, Norway, and Sweden, there has clearly been an overrepresentation of BCSs in the southern urban areas of the country, especially around university hospitals. On the other hand, a large part of the total population lives in these geographically limited but more densely populated areas; for instance, over 1/3 of the Danish population live in the Copenhagen metropolitan area, which is the best represented region in the Nordic BCSs. The populations of the large metropolitan areas are usually more varied in terms of socio-economic conditions and ethnicity than rural and small-town populations. While there are some areas in Finland (such as South-Eastern Finland) and Sweden (in Northern Sweden) that are not covered by any cohorts, the studies represent both urban and rural, and wealthy and disadvantaged areas. The same applies to the other Nordic countries, including Iceland, whose only study is nationwide. Especially in Sweden, the geographical scope of the birth cohort studies has clearly become more varied after the first decades.

**Figure 4.**
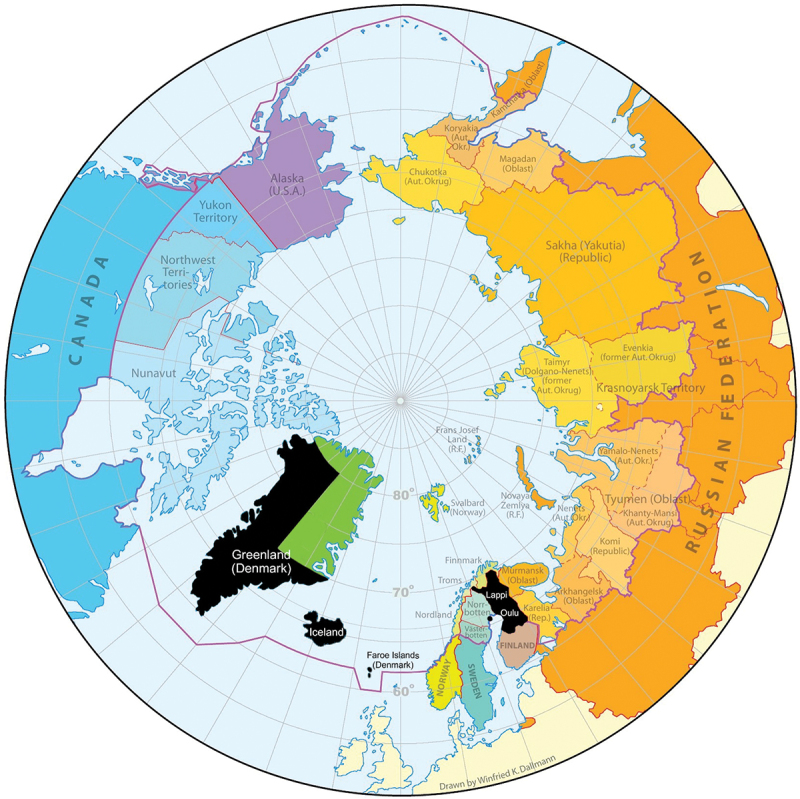
Map of the Arctic administrative areas and the Nordic birth cohorts located in the Arctic area (marked as black).^6^

Eight BCSs were from Arctic regions, and their topics reflect the specificities of these areas (Figure 4). The first Finnish birth cohort study (F1) focused on the disadvantaged Northern and Arctic areas, and specifically addressed the regional health inequalities in the country, initially in relation to perinatal health and mortality. Two other Finnish birth cohorts (F7, F15) are located in the same area. The only Arctic birth cohort of Sweden (S23) took place in Luleå, focusing on environmental exposures. Among Danish cohorts, D7 is located in Faroe Islands and focuses on Arctic livelihoods, particularly on seafood diet and marine contaminants. Two Greenlandic BCs focus on Greenlandic Inuit health. Indigenous people also participate in birth cohort studies outside their traditional settlement area. For instance, there are large populations of Sámi people in the Southern and especially in the capital areas of Norway, Sweden, and Finland.

### Eligibility criteria for participants

The general inclusion criterion for a birth cohort participation is being born or being a pregnant mother at a certain time in a certain geographical area. However, not all the children all mothers meeting these criteria participate in the studies either because they or their parents decline or because they are not eligible. Of the reviewed studies, 61% announced one or more specific exclusion criteria (see Table 1), insufficient language skills (*n* = 26, a third of all studies) being the most common criterion. In these studies, a parent, usually the mother, or parents needed to be able to understand the official language of the country well enough to answer questionnaires or participate in interviews. Another common criterion was the health of the mother, the baby, or both. Preterm babies, and babies with severe malfunctions or other severe diseases were often excluded from the study. On the other hand, we also identified 12 at-risk cohorts with a special focus on children with genetic or other risk factors, such as preterm birth (F15). Healthy babies without the specified risk factor(s) were excluded from these studies.

Five studies excluded potential participants because of their ethnic background. The Copenhagen mother-child cohort (D11), established in 1996, required that both the parents and grandparents of the unborn child were born and raised in Denmark; the Ivaaq cohort (D16) and ACCEPT cohort (D21) examined only ethnic Greenlanders; the GLAKU BCS (F11) excluded participants of non-Finnish origin; and the MIDIA BCS (N6) excluded participants of non-Caucasian origin, because the examined risk gene for diabetes is lacking in non-Caucasians.

All the cohorts examining childhood diseases and conditions included children to the study regardless of gender whereas some of the retrospective cohort studies recruiting adults were single-gender cohorts, including only women (S4) or only men (D3, S1, S6, S9). One cohort (S21) studying perinatal depression used an age criterion and excluded mothers under the age of 18. Some studies have excluded twin pregnancies or subsequent babies from the same mother (e.g. D8, D16, D22, F11, F20, S3, S10).

### Types of data collected

In our review, we included studies which have collected two or more types of data within a study programme. Questionnaires, surveys, or interviews were included in all studies. Questionnaires were included in 76, and interviews in 32 studies. Biological data such as blood samples were the second most common data types across the birth cohort studies (included in 73% of the studies). Register data was also commonly utilised (in 65% of the studies). Clinical data, which often includes multiple different tests, such as rest-ECG, skin allergy tests, spirometry, physical activity tests, eye examinations, dental examinations, or body composition tests (see for instance [45,46] were collected in 51 (65%) of the cohort studies. Psychological, psychiatric, neurological, or cognitive evaluations were less common but were included altogether in one third of the studies (*n* = 28, 35%). It should be noted that many of the study programmes also initiated sub-studies with smaller sub-samples of research participants, which we have not systematically reviewed. In these sub-studies, more varied data collection methods were commonly utilised.^6^^6^
Original map courtesy of Winfried K. Dallmann, Norwegian Polar Institute.

Fifteen of the reviewed BCSs collected exceptionally versatile data, representing five out of the six data type categories of this review (types 1–5, see Appendix 2). These studies were mostly medium-sized general mother-child birth cohorts with multiple follow-ups over the years. However, environmental data (type 6), such as house dust, was not included in these studies, and was rarely collected in the birth cohort studies in general.

### Participants and knowledge producers

The active participants of the birth cohort studies were commonly the mother and her child, i.e. the index child. Fathers also participated in many studies, but not to the same extent as mothers. In all the traditional BCSs where the participants were recruited either during pregnancy or right after birth, mothers responded to questionnaires/interviews, and fathers/partners were given the possibility to also respond in little over half of them (66%, *n* = 37). In these types of studies, the index child rarely responded to the questionnaires/interviews themselves (33%, *n* = 19), and parent(s) responded about their own and their child’s health. Naturally, this has to do with the age of the index children; they usually started to respond to the surveys only as teenagers, and many Nordic birth cohorts are not that mature yet. In those BCSs where recruitment happened years or decades after the birth, “index children” responded to questionnaires in nearly all studies (96%, *n* = 22). Recently, grandparents have been invited to participate in birth cohort studies that have been established decades earlier (for example in S13 initiated in 1996, F1 initiated in 1965), although their participation is still rare in Nordic BCSs. Healthcare professionals were respondents in one fourth of all BCSs (*n* = 25, 32%), and school teachers responded in 9 studies. No clear temporal trends regarding participant groups were observed. That is, for instance, fathers were as often included in the studies established in the 1960s and in 2000s.

## Discussion

This review is the first to summarise and describe the basic characteristics of birth cohort studies established in Nordic countries. Over the course of several decades, from the 1950s to the 2020s, 79 Nordic birth cohort studies have produced important knowledge especially on the effects of early life exposures on health later in life, but also on other topics such as the ageing process, mental health, and behavioural and social issues. Even though no new birth cohort studies have been established in recent years, more than half of the reviewed birth cohort studies are still ongoing. Our review was able to detect a considerably greater number of Nordic mother/parent-child birth cohorts than previous reviews on European BCSs [5,6], and also included BCSs where recruitment happened later in life.

### Hundreds of thousands of Nordic ‘index children’

Over 600,000 Nordic inhabitants have participated in the 79 birth cohort studies as index children, and, on top of that, several hundred thousand parents have also done so. Recently, grandparents of the index children have been included in some of the Nordic birth cohort studies as well. The participant numbers are high with respect to the total population of the five Nordic countries, which is approximately 27 million. For instance, in Australia and New Zealand, an area with a comparable population, Townsend et al. [15], identified 23 birth cohort studies with less than 40,000 index children.

The general societal conditions and practices in the Nordic countries provide explanations for the prominence of BCSs in this area. As wealthy welfare societies they have had sufficient funds to facilitate large scale longitudinal study programmes [47–49]. They also have a long tradition of monitoring the health of pregnant women and children as well as giving birth in hospital settings. These facilitate contacting prospective participants and gathering data. Moreover, tracking down cohort members to follow-ups is enabled by existing national registers, citizen databases, and personal identification numbers [28].

#### Regional and ethnic representativeness

Despite the large surface area of the Nordic countries and some overrepresentation of large cities, different geographical areas are quite well represented in the Nordic birth cohort studies, at least in proportion to their population. Only seven nationwide birth cohort studies (none in Finland or Sweden) have been conducted, but wide geographical areas are still covered by many regional birth cohorts as well. These areas include both wealthy and disadvantaged and urban and rural areas.

There have also been birth cohort studies in areas that are inhabited with substantial minority populations, such as the traditional settlement areas of the Sámi people. As there might be some specific health and wellbeing issues among indigenous peoples, it is valuable that BC data is produced among them as well. However, since the Nordic countries do not collect data on ethnicity in large-scale or population based surveys [38,50], the health problems of the minorities do not become visible even when the minorities are included in these studies. Sometimes, this problem is partially circumvented by using measures such as the place of birth, mother tongue, or nationality in data sweeps [49]. Several Nordic birth cohorts include health information on the indigenous peoples, and this information could benefit health research and promotion.

Even though only a few Nordic birth cohort studies announced ethnic exclusion criteria, the more common language criterion has reduced the possibilities of other ethnic minorities, especially newly arrived immigrants, to participate in birth cohort studies. While the language criterion is understandable for practical reasons, it easily leaves disadvantaged parts of the population out of the study scope. Birth cohort studies could offer an opportunity to disentangle the underlying mechanisms of health inequalities regarding ethnic, migration or minority background, if this information was collected. Grosser and colleagues [49] have reviewed European birth cohort studies from this perspective and criticised them for wasting the opportunity to address health inequalities. Moreover, based on earlier research, ethnic minorities, people in disadvantaged socio-economic positions and those at greater risk of developing health problems tend to participate in this type of studies less often and are more likely to drop out [51]. This type of attrition can cause systematic biasin the data collection and limit the generalisability of findings [4,52].

Oversampling of ethnic minority groups has been suggested as a way to address the mentioned systematic biases [49]. Even though this is not simple because of the lack of data on ethnicity, some Nordic birth cohort studies have addressed these questions. For example, the Danish Longitudinal Survey of Children Born in 1995 (DALSC) and the Odense Child Cohort have made special efforts to recruit participants with non-western backgrounds. Since the Nordic countries face relatively large health inequalities despite their welfare model [37,53], both socio-economic and ethnic variables should preferably be addressed. However, as this is a sensitive and complex matter, it requires taking various and conflicting ethical aspects into consideration.

#### From preterm birth to allergy studies

The findings of this review evidence a strong medical focus and, more specifically, a “Developmental Origins of Health and Disease” approach (DOHaD, see [13; 15] in the Nordic birth cohort studies. Approximately a third of the mother/parent-child cohorts, and a fifth of the studies which recruited index children later in life had a general focus covering a range of exposures to child development, health, and well-being while the remaining studies addressed more specific topics, often still within the DOHaD frame. A similar trend between specific and general cohort types has been detected in previous birth cohort reviews [1,15]. Many Nordic studies started with a specific focus, but extended it over the course of the study, a phenomenon also common in birth cohort studies elsewhere [15].

The cohort studies conducted in different geographical areas reflect, to some extent, their specific disease burden and conditions. For instance, the birth cohort studies conducted in South-East Asia and Eastern Mediterranean regions have focused on growth, mortality, nutrition, and infectious diseases, and less often on non-communicable diseases [54], whereas European birth cohort studies have predominantly concentrated on nutrition-related, neuropsychological, and disease-specific outcomes, and exposures related to lifestyles or the physical and social environments [49]. Based on the findings of this review, Nordic birth cohorts with a specific focus have studied non-communicable diseases that are common in the region. Particularly common were birth cohort studies focusing on asthma, allergy, and other atopic diseases, which are among the most common chronic diseases among children in many industrialised countries (see [55]. The incidence of different atopic diseases has increased in the last decades, and longitudinal epidemiological birth cohort study is an optimal research model for detecting cause-effect relationships in their development [22]. In addition, data on allergies are not fully covered by available registers since they are health conditions that often do not result in hospital contact [56], which makes the collection of data in BCS especially valuable. Three of the Nordic BCSs also focus on type 1 diabetes, which has exceptionally high prevalence in Norway, Finland, and Sweden [57].

The foci of the study programmes have changed over time, which likely reflects the rise of the standard of living and changes in disease prevalence but also the cumulation of medical knowledge on certain topics and risk factors. Based on this review, many of the earliest Nordic birth cohort studies were centred on conditions with severe or even lethal consequences, such as risk factors of preterm birth and cardiovascular diseases. This review shows, similarly to other European birth cohort studies [22], that in the 1990s, atopic diseases became more prevalent topics.

When a study focuses purely on medical/clinical conditions, it may miss out the opportunity to study social determinants of diseases (see [49]) and wellbeing in general. Since BCSs with a medical scope commonly include questionnaires addressing a wide variety of issues, such as social situation, wealth, and occupation, there is clearly a lot of potential to use birth cohort data in other disciplines, too. Somewhat surprisingly, none of the Nordic birth cohorts had an educational focus, whereas for instance in the UK [58], long-term birth cohorts such as the Next Step study [59] specifically include variables exploring the educational realm. Education and schooling are instrumental for personal, social, political, and cultural development [60], and birth cohorts with a focus on educational settings, learning, school performance, educational transitions, and future adult health and wellbeing would be important to conduct in Nordic contexts as well.

#### Respondents and knowledge production

The birth cohort studies have been immensely valuable, as they focus on the underrepresented groups in medical research – mothers, infants, and young children [1,61]. Medical treatment is largely based on knowledge produced on research on men, which is a pitfall and an obstacle also for gender equity in healthcare [62]. On the other hand, men as fathers, in relation to their children, has not been a popular topic of medical research [63], and while birth cohort studies include fathers as participants, they have partially missed their opportunity to address the issue. According to our review, over half of the Nordic birth cohort studies collected data from fathers while all mothers were respondents, a finding similar to a previous Australian and New Zealand review [15]. In addition, fathers are not as active as mothers in responding to surveys/questionnaires, even when given a chance (f. ex [64]. The absence of fathers in birth cohort studies was discussed already in the 1990s [18], but no substantial progress has been seen regarding their involvement. The traditional family norm of two-parent families with mother and father has been a given in the older birth cohort studies. In future birth cohort studies, more inclusivity when addressing participants is called for.

Even though index children are the central participants in birth cohorts, they have seldom been active respondents in surveys or interviews regarding their own health and wellbeing for the first 10–15 years of the study. Clinical and biological data have been collected with young children, but self-reports have not. The tendency of adults to ignore children’s views exists in many research fields and study types, and as seen, also in birth cohort studies. Often scientists explore children’s experiences through the accounts provided by adults – parents, healthcare professionals, or teachers – rather than listen to children’s own accounts of their life worlds [65–67]. When it comes to medical topics, parents are likely to be able to provide information on their child’s health issues when responding to surveys, but adult ’proxies’ are not necessarily able to give valid accounts on children’s social or emotional lives, which are often central to health and wellbeing (see [67]. This is a shortcoming also from the perspective of children’s rights and research ethics [68].

#### Limitations

This review aimed to chart and review Nordic birth cohort studies from their first initiation until the spring of 2022. Our review was limited to information found in abstracts, published articles, cohort profile articles, official cohort webpages as well as cohort databases. It is possible that we have missed some birth cohort studies that would fulfil our inclusion criteria. We did not get in touch with cohort leads, like many previous birth cohort study reviews have done (e.g [1]. Thus, the information we have collected from the birth cohort studies is somewhat limited, especially regarding most recent developments and the sub-study data. However, as our aim was to offer a basic description of the main data collection phases of the studies and how the cohort characteristics have evolved over time, the information found in the aforementioned sources can be considered sufficient. We excluded birth cohort studies which cover areas larger than a nation state. For instance, the Finnish study LUKAS, which is part of a European birth cohort PASTURE [69], was excluded, similarly to INUENDO, which include Greenlandic, Ukrainian, and Polish participants [70]. Moreover, setting the limit to a minimum of 200 participants have left out some interesting and unique birth cohorts, such as the Arctic Sámi Birth Cohort [71] and Icelandic Citizens born in 1987 [72].

## Conclusions

Nordic countries have hosted 79 longitudinal birth cohort studies with more than 600,000 index children. These studies have provided valuable scientific contributions in many fields, especially in epidemiology and medical sciences. Our review provided an extensive overall description and insights on the nature of the Nordic birth cohort studies, and the development of this research tradition over time. This type of information is useful when new birth cohorts are planned and established in Europe, and when cross-cohort collaboration is initiated. Indeed, many of the ongoing and some terminated studies offer possibilities for secondary analysis of data. This massive data pool could also be used more extensively in non-medical disciplines, such as social sciences, gender studies, education, and humanities, which in turn would widen the scope of knowledge production on birth cohort data.

In recent years, the Nordic countries have not been active in launching new birth cohort studies. Many BCSs have shown agility in adjusting and responding to the changing world; for instance, some BCSs have included COVID-19 related data collection to their data sweeps. Yet, the launch of new, generally aimed birth cohort studies would help understand the challenges babies born in the 2020s will be facing as they live in a very different environment than the older generations. In addition to commonly collected data from mothers and index children, we would encourage to include more versatile data including data from fathers or other primary caregivers and grandparents and questionnaire/interview data from young children. Furthermore, Nordic birth cohorts offer possibilities for indigenous and minority research, even though the laws regarding asking ethnicity in surveys in the Nordics limits these possibilities. These research directions could be utilised more in the existing Nordic birth cohort data, work that should be done in close collaboration with indigenous and other minority communities. Very few circumpolar birth cohorts exist outside Nordic countries [73]. For instance, an inventory of Canadian birth cohorts reviewed 46 cohorts and only two of them (4.7%) were Arctic [17], the Nunavik Child Development Study (NCDS) being one of them [74]. Cross-cohort comparisons between Arctic birth cohort studies and embarking new Arctic studies could greatly benefit knowledge production on health and wellbeing of the Arctic populations.

We recognise the challenges that the large birth cohort studies have faced in the first decades of the 2000s, such as the descendent willingness to participate in such studies and the stricter regulation of the data gathering and sharing processes, as well as related expenses. In the future, it would be worthwhile to study trends in science politics and financing to provide insights in how they direct the planning and execution of BCSs [75–78].

## Appendices

To identify relevant original research articles, we conducted searches from EBSCO, ProQuest, and Scopus databases.

**EBSCO and ProQuest** include several databases which are listed in Table A1.

**Table A1.** Databases included in EBSCO and ProQuest.

EBSCO	ProQuest		
Academic Search Ultimate	ABI/INFORM Collection‎ (1971 - current)		
America: History & Life	ABI/INFORM Dateline (1985 - current)		
APA PsycArticles	ABI/INFORM Global		
Arctic & Antarctic Regions	ABI/INFORM Trade & Industry (1971 - current)		
Art & Architecture Complete	Accounting, Tax & Banking Collection‎ (1971 - current)		
Audiobook Collection (EBSCOhost)	Applied Social Sciences Index & Abstracts (ASSIA)‎ (1987 - current)		
Avery Index to Architectural Periodicals	Asian & European Business Collection‎ (1971 - current)		
Business Source Ultimate	Business Market Research Collection‎ (1986 - current)		
CINAHL with Full Text	Canadian Business & Current Affairs Database‎		
Communication & Mass Media Complete	Coronavirus Research Database‎		
eBook Academic Collection (EBSCOhost)	Criminology Collection (1975 – current)		
eBook Collection (EBSCOhost)	Early English Books Online		
EconLit with Full Text	Early Modern Books‎		
ERIC	Ebook Central‎		
GeoRef In Process	Education Collection (1966 - current)		
GeoRef	Education Database‎ (1988 - current)		
GreenFILE	ERIC‎ (1966 - current)		
Historical Abstracts with Full Text	International Bibliography of the Social Sciences (IBSS) (1951 - current)		
Library, Information Science & Technology Abstracts	Library & Information Science Abstracts (LISA)‎ (1969 - current)		
MEDLINE	Library & Information Science Collection (1969 - current)		
MLA Directory of Periodicals	Library Science Database‎ (1970 - current)		
MLA International Bibliography	Linguistics and Language Behaviour Abstracts (LLBA)‎ (1973 - current)		
OpenDissertations	Linguistics Collection (1973 - current)		
Regional Business News	Linguistics Database‎		
Teacher Reference Center	PAIS Index‎ (1914 - current)		
	Policy File Index‎ (1990 - current)		
	Political Science Database‎ (1985 - current)		
	Politics Collection (1914 - current)		
	Publicly Available Content Database‎		
	Social Science Database information		
	Social Science Premium Collection‎ (1914 - current)		
	Sociological Abstracts‎ (1952 - current)		
	Sociology Collection (1952 - current)		
	Sociology Database‎ (1985 - current)		
	Worldwide Political Science Abstracts‎ (1975 - current)		

The following search strategies were used without imposing language or publication status restrictions. However, the search was restricted to peer-reviewed research articles in EBSCO and ProQuest.

- **EBSCO** (all databases) in 26th of May 2020 and update search in 16th of May 2022; abstract search [covers titles & keywords]: “birth cohort*” AND (Finland OR Finnish OR Sweden OR Swedish OR Norway OR Norwegian OR Denmark OR Danish OR Iceland OR Icelandic)
- **ProQuest** (all databases) 27th of May 2020 and update search 16th of May 2022; abstract search [covers titles]: “birth cohort*” AND (Finland OR Finnish OR Sweden OR Swedish OR Norway OR Norwegian OR Denmark OR Danish OR Iceland OR Icelandic)
- **SCOPUS** 27th of May 2020 and update search in 16th of May 2022; title, abstract and keyword search: “birth cohort*” AND (Finland OR Finnish OR Sweden OR Swedish OR Norway OR Norwegian OR Denmark OR Danish OR Iceland OR Icelandic

The review is not yet registered. The review protocol can be accessed from the authors.

## Appendix 2: The characteristics of the included birth cohorts

**Table:**

Country, Code, Study name^1^	Timing	Region	No. of index child-ren	Initial topic, general/specific	Retrospective/Prospective, recruitment age	Follow-ups 1. Below age of 1 2. Ages 1–6 3. 7–14 4. 15–29 5. Later					Types of data 1. Biological 2. Clinical/physical examination 3. Questionnaire/ Interviews (q/i) 4. Psychological/psychiatric/neurological/cognitive test/evaluation 5. Registers 6. Environmental						Respondents on questionnaires/interviews 1. Mother 2. Father 3. “Index child” 4. Healthcare professional 5. Teacher^2^					Exclusion/specific inclusion criteria 1. Non-sufficient language proficiency (excl) 2. Non-sufficient health (excl) 3. At risk (incl) 4. Sex, Ethnicity, Age (excl)			
**DENMARK**						1	2	3	4	5	1	2	3	4	5	6	1	2	3	4	5	1	2	3	4
D1 Copenhagen Perinatal BC	1959–	Copenhagen	9182	connections between factors early in the life, risk factors central in life and health status, **General**	Prospective Pregnancy	x	x			x		x	q	x	x		x		x					x	
D2 The 1897, 1914 and 1936 BCs	1964–	Copenhagen County	1052	coronary heart disease, **Specific**	Retrospective & prospective 70, 50, 40					x	x	x	qi	x	x				x						
D3 Danish Metropolitan 1953 BC	1965– 2016	Copenhagen metropolitan area	11532	intergenerational mobility and differential life-chances, **Specific**	Retrospective & prospective 12			x	x	x	x	x	qi	x	x		x		x	x	x				s
D4 Viborg BC	1983–1988	Viborg	1193	development of allergy, **Specific**	Prospective Postnatal		x				x	x	q				x	x							
D5 Healthy Habits for Two (HHf2)	1984-	Odense and Aalborg	12000	health behaviour during pregnancy, **General**	Prospective Pregnancy	x		x	x			x	q		x		x		x						
D6 Odense 1985 BC	1985–2011	Odense	276	development of asthma and allergic diseases, **Specific**	Prospective Postnatal	x	x	x	x		x	x	i				x	x							
D7 Faroese BCs (Chef 1–5)	1986–	Tórshavn and Faroese Islands Arctic	approx.2500	effects of seafood diet and marine contaminants on growth and development, **Specific**	Prospective Postnatal	x	x	x	x		x	x	qi	x	x		x	x					x		
D8 The Danish Foetal Origins Study 1988 (DaFO88)	1988–2009	Aarhus	965	effects of early life exposures on pregnancy outcomes, **General**	Prospective Pregnancy	x			x		x		qi		x		x								
D9 Aarhus BC	1989–	Skejby	100000	risk factors for pre-term birth, **Specific**	Prospective Pregnancy	x	x				x		q		x		x					x			
D10 The 1895,1905,1910 and 1915 Danish BCSs	1995–2016	Denmark	4326	health and functioning of the very old, **General**	Retrospective & Prospective Ages 92–100					x	x	x	i	x	x				x						
D11 The Copenhagen mother-child cohort (COP1, COP2)	1996–	Cop 1: 3 hospitals, Cop2: 2 university hospitals	2988	mechanisms of human growth and reproduction, **Specific**	Prospective Pregnancy	x	x	x	x		x	x	q				x	x				x	x		e
D12 Danish National BC	1996–	Whole country	100000	effects of early life exposures on disease, **General**	Prospective Pregnancy	x	x	x	x		x		qi		x		x	x	x			x			
D13 The Danish Longitudinal Survey of Children born in 1995 (DALSC)	1996–	Denmark	6011	children’s growing-up conditions in contemporary society, **General**	Prospective Postnatal	x	x	x	x				qi		x		x	x		x					
D14 Copenhagen Prospective Studies on Asthma in Childhood COPSAC 2000	1998–2016	Greater Copenhagen area	411	gene-environment interactions in atopic diseases, **Specific**	Prospective Postnatal	x	x	x	x		x	x	q			x	x	x				x	x	x	
D15 The DARC BCS	1998–2013	Odense	562	atopic and allergic diseases in children, **Specific**	Prospective Postnatal	x	x	x			x	x	i			x	x	x				x	x		
D16 Ivaaq cohort	1999–2012	Nuuk, Maniitsoq, Ilulissa, Greenland Arctic	403	effects of selected exposures on pregnancy outcome and child development, **General**	Prospective Pregnancy	x	x	x			x		qi				x								e
D17 Copenhagen Child Cohort (CCC 2000)	2000–	Copenhagen area	6090	developmental epidemiology of mental health problems and psychopathology, **Specific**	Prospective Postnatal	x	x	x	x		x	x	qi	x	x		x	x	x	x	x				
D18 West Jutland Cohort	2004–	County of Ringkjøbing	3681	effects of inequalities and social disparities on health and well-being, **Specific**	Prospective & retrospective 14–15				x	x			qi		x				x						
D19 COPSAC 2010	2008–	Copenhagen	700	asthma and other wheezy disorders, eczema and allergy, **Specific**	Prospective Postnatal	x		x	x		x	x	qi			x	x	x					x		
D20 Odense Child Cohort	2010–	Odense	2549	interaction between social, biological and environmental determinants of health, **General**	Prospective Pregnancy	x	x	x	x		x	x	q		x		x	x	x						
D21 Adaptation to Climate Change, Environmental pollution, and Dietary Transition (ACCEPT) birth cohort	2010-	Greenland, 16 towns in North, Disko Bay, West, South and East Arctic	587	environmental exposures during pregnancy, dietary changes and health during a period of lifestyle transition and climate change. **Specific**	Prospective Pregnancy	x	x				x		qi				x	x		x					x
D22 Barrier dysfunction in Atopic newBorns studY (BABY)	2017–	Rigshospitalet, Copenhagen and Nordsjællands Hospital, Hillerød	450	the development of atopic dermatitis, **Specific**	Prospective Pregnancy	x	x				x	x	qi		x		x	x				x	x	x	
**FINLAND**						1	2	3	4	5	1	2	3	4	5	6	1	2	3	4	5	1	2	3	4
F1 Northern Finland BC 1966 (NFBC1966)	1965–	Lappi, Kainuu, Northern Ostrobothnia regions Arctic	12231	risk factors involved in pre-term birth and intrauterine growth retardation, **Specific**	Prospective Pregnancy	x	x	x		x	x	x	q	x	x		x	x	x	x	x				
F2 PLASTICITY – life long follow-up of cognitive ability after birth risks	1971–	Helsinki metropolitan area	1196	connection between birth risks and cognitive abilities and health, **General**	Prospective Postnatal		x	x	x	x	x	x	q	x	x		x		x	x			x	x	
F3 Helsinki longitudinal temperament cohort	1975–2005	Helsinki	6468	infant temperament, effects of maternal subjective stress during pregnancy, **Specific**	Prospective Pregnancy	x	x	x					q		x		x								
F4 The cardiovascular risk in young Finns Study (Laseri)	1980–	Finland, Helsinki, Kuopio, Oulu, Tampere, Turku	3596	levels of CHD risk factors and their determinants in children and adolescents, **Specific**	Prospective & Retrospective 3,6,9,12,15,18		x	x	x	x	x	x	q	x			x	x	x						
F5 From childhood to teenage years	1981–1997	Finland, rural community in Eastern Finland	205	systolic blood pressure during childhood, **Specific**	Prospective, 6 months	x	x	x	x			x	q		x		x	x	x						
F6 Arvo Ylppö Longitudinal Study (AYLS)	1985–2012	Uusimaa area	2193	perinatal risk factors, developmental disorders, behavioural & cognitive problems, **General**	Prospective Postnatal	x	x		x			x	qi	x	x		x	x		x				x	
F7 Northern Finland BC 1986 (NFBC1986)	1985–	Lappi, Kainuu, Northern Ostrobothnia regions Arctic	9432	genetic, biological, social and behavioural risk factors of diseases, **General**	Prospective Pregnancy	x	x	x	x	x	x	x	q	x	x		x	x	x	x	x				
F8 Finnish 1981 Nationwide BCS	1989–2005	Helsinki, Kuopio, Oulu, Tampere, Turku	5813	childhood mental and behavioural problems and long-term outcomes, **Specific**	Prospective & Retrospective Age 8			x	x				qi		x		x	x	x		x	x			
F9 Diabetes Prediction and Prevention Project (DIPP)	1994–2011	Turku, Oulu, Tampere	17000	prenatal and childhood diet, connection to type 1 diabetes, allergies, asthma, obesity, **Specific**	Prospective Postnatal	x	x	x	x		x		qi		x		x	x					x	x	
F10 Helsinki BCS 1934–44, 1924–1933, (Idefix)	1995–	Helsinki	15846	importance of early life factors on later health outcomes, **General**	Retrospective & prospective 51–71					x	x	x	q		x				x						
F11 Glycyrrhizin in Liquorice (GLAKU) cohort	1998–	Helsinki	1049	effects of maternal liquorice consumption on developmental outcomes of offspring, **Specific**	Prospective Postnatal	x		x	x		x	x	q	x	x		x		x				x		e
F12 Development and Functioning of Very Low Birthweight Infants from Infancy to School Age (PIPARI)	2001–	Turku	478	effects of foetal risk factors on long-term outcome, neonatal intensive care outcome, **General**	Prospective Postnatal	x	x	x	x		x	x	q	x			x	x	x	x		x	x	x	
F13 Helsinki studies of very low birth weight adults	2004–	Finland, Uusimaa	338	how being born preterm at very low birth weight affects adult health and well-being, **Specific**	Prospective & retrospective, 19–26				x	x	x	x	q	x					x					x	
F14 Steps to the Healthy Development and Well-being of Children (STEPS) Study	2007–	Southwest Finland hospital district	1827	early development, health and well-being of children, effects later in life, **General**	Prospective Pregnancy	x	x	x			x	x	q	x	x		x	x			x	x	x		
F15 Preterm Birth, Pregnancy and Offspring Health in Adult Life (ESTER)	2009–	Lappi, Kainuu, Northern Ostrobothnia areas Arctic	779	effects of preterm birth, maternal hyper-tension, and gestational diabetes, **Specific**	Retrospective & prospective 20–24				x		x	x	q		x				x				x	x	
F16 CHILD SLEEP BC	2011–2015	Tampere	1673	sleep in families with newborn children, **Specific**	Prospective Pregnancy	x	x				x		q		x		x	x				x			
F17 FinnBrain BCS	2011–	Turku & Åland Islands	3837	effects of early life stress (including prenatal) on child brain development, **Specific**	Prospective Pregnancy	x	x	x			x	x	q	x	x		x	x	x	x		x	x		
F18 Kuopio BC (KuBiCo)	2012–	Kuopio	10 000	effects of genetics, epigenetics, and risk factors during pregnancy on the somatic and psychological health status of the mother and the child, **General**	Prospective Pregnancy	x	x	x			x		q		x	x	x								
F19 Infants born at the Central Finland Central Hospital	2014–2018	Finland, Central Finland	212	foetal microbiome, association with colic and atopic manifestations, **Specific**	Prospective, Pregnancy	x	x				x		q		x		x			x			x		
F20 Health and Early Life Microbia (HELMI)	2016–	Helsinki region	1055	intestinal microbiota development, lifestyle, environmental, and genetic factors, **Specific**	Prospective Pregnancy	x	x				x		q	x	x	x	x	x		x		x	x		
**NORWAY**						1	2	3	4	5	1	2	3	4	5	6	1	2	3	4	5	1	2	3	4
N1 Bergen 1940 BC	1970–1992	Bergen	1331	conditions of work-insufficiency, medical, psychological, educational, **Specific**	Retrospective & prospective 30			x	x	x		x	qi	x	x				x	x				x	
N2 Nord‐Trøndelag 1973, 1983, 1994, 2006 cohorts	1973–2006	Nord- Trøndelag	1800	oral health, connection with social status, **Specific**	Prospective & Retrospective 13–14, 35–44			x	x	x		x	q						x	x					
N3 Patterns of sugar consumption in early childhood	1979-	Farming town	231	sugar consumption and dietary habits, **Specific**	Prospective Pregnancy	x	x	x					qi				x			x					
N4 Environment and Childhood Asthma birth cohort study (ECA)	1992–2009	Oslo	3754	air pollution in relation to asthma in young children, **Specific**	Prospective Postnatal	x	x	x	x		x	x	qi			x	x	x				x	x		
N5 Norwegian Mother, Father and Child Cohort Study (MoBa)	1999–	Whole country	114000	aetiology of complex diseases, exposures and lifestyles during pregnancy, **General**	Prospective Pregnancy	x	x	x			x	x	q		x		x	x	x	x		x			
N6 Environmental causes of type 1 diabetes (MIDIA)	2001–	Whole country	900	environmental causes of type 1 diabetes, **Specific**	Prospective Postnatal	x	x	x	x		x	x	q				x	x					x	x	e
N7 Human Milk Study (HUMIS)	2002–	Whole country	2606	organic pollutants in mothers’ milk, exposure factors and health outcomes, **Specific**	Prospective Postnatal	x	x	x			x		q		x		x					x			
N8 Norwegian Microbiota Cohort (NoMIC)	2002–	Southern Norway	601	gut microbiota development, composition in connection to health, **Specific**	Prospective Postnatal	x	x	x			x	x	qi				x		x			x			
N9 The Trondheim Early Secure Study (TESS)	2007–	Trondheim	1250	mental health, psychosocial development, weight, and health related behaviour, **Specific**	Prospective & Retrospective 4		x	x			x	x	qi	x	x		x	x	x	x	x	x			
N10 Akershus Birth Cohort Study ABC	2008–2013	Akerhus	3752	postpartum mental health, **Specific**	Prospective Pregnancy	x	x						q		x		x					x			
N11 The Norwegian Influenza Pregnancy Cohort (NorFlu)	2010-	Oslo, Bergen	4500	consequences of influenza A (H1N1) during pregnancy, **Specific**	Prospective Pregnancy	x	x	x			x	x	q	x	x		x			x	x			x	
N12 The Akershus Cardiac Examination ACE 1950 study	2011-	Akerhus	3706	the development and progression of cardiovascular and cerebrovascular disease (CVD/CeVD), **Specific**	Prospective & retrospective 63–65					x	x	x	qi	x					x						
**SWEDEN**						1	2	3	4	5	1	2	3	4	5	6	1	2	3	4	5	1	2	3	4
S1 The study of men born in 1913 and 1943	1963–2014	Gothenburg	1653	risk factors for cardiovascular disease (CVD) among men, **Specific**	Prospective & Retrospective 50					x	x	x	qi		x				x						s
S2 Stockholm metropolitan study (1953 cohort)	1963–1986	Stockholm Metropolitan area	13476	intergenerational mobility, deviance, marital adjustment, **Specific**	Prospective & Retrospective 12/13			x	x	x			q/ i	x	x		x		x			x			
S3 The Helsingborg Birth Cohort (HbgBC)	1964–	Helsingborg	4091	future health of mothers and offspring, effects of smoking, **Specific**	Prospective Pregnancy	x			x	x		x	q		x		x		x	x					
S4 The Prospective Population Study on Women in Gothenburg (PPSW)	1968–2011	Gothenburg	1462	CVDs, diabetes, cancer, dementia, mental illness in middle aged women, **Specific**	Prospective & Retrospective 38, 46, 50, 60					x		x	qi	x	x				x	x					s
S5 Growing up in Uppsala	1969–1983	Uppsala	1715	role of public services in identification and treatment of health and adjustment problems, **Specific**	Prospective & Retrospective 4		x	x	x			x	qi	x	x		x	x		x	x				
S6 Uppsala Longitudinal study of adult men (ULSAM)	1970-	Uppsala	2322	identifying metabolic risk factors for cardiovascular disease, **Specific**	Prospective & retrospective, 50					x	x	x	qi	x					x						s
S7 Gothenburg H70 study	1971–	Gothenburg	4853	normal ageing processes, social and medical conditions, **General**	Prospective & Retrospective 70					x	x	x	qi	x	x				x	x		x			
S8 Swedish asthma and allergy birth cohort	1974–2007	Linköping county	1701	development of asthma and allergic diseases, **Specific**	Prospective Postnatal		x	x		x			qi		x		x	x	x						
S9 The coronary risk factor study in southern Sweden (CRISS)	1990–1997	Helsingborg	991	coronary risk factors among middle aged men, **Specific**	Prospective & Retrospective 37					x	x	x	q						x						s
S10 Children, Allergy, Milieu, Stockholm, Epidemiology study (BAMSE)	1994–	Stockholm	4089	risk factors for asthma, allergic diseases and lung function, **Specific**	Prospective Postnatal		x	x	x		x	x	q		x		x	x	x			x	x		
S11 South East Sweden BCS (SESBiC)	1995–2009	Hässleholm, Western Blekinge	1723	children’s mental development, risk, resilience, psychosocially burdened families, **Specific**	Prospective Postnatal		x	x					q		x		x		x						
S12 Paediatric Allergy Study (The BAS cohort)	1996–2010	Jämtland	1231	development of allergy during childhood, **Specific**	Prospective Postnatal		x	x			x		q				x	x							
S13 N5 Gotland 1989 birth cohort	1996–	Isle of Gotland	707	respiratory allergy, eczema and atopic sensitisation, **Specific**	Retrospective & prospective 7–8			x			x		q				x	x	x						
S14 All Babies in Southeast Sweden (ABIS)	1997–	Southeast Sweden	17055	diabetes type 1, immune-mediated diseases (allergy, asthma, IBD, coeliac disease, cancer, etc., **Specific**	Prospective Postnatal		x	x			x		q		x		x	x	x						
S15 Diabetes Prediction in Skåne (DiPiS)	2000–2019	Skåne	3868	genetic and immunologic markers predicting type I diabetes, and associated immune mediated diseases, **Specific**	Prospective Postnatal	x	x	x	x		x	x	q				x	x						x	
S16 Good ageing in Skåne (GÅS-SNAC)	2003–	Skåne	4500	health, illness, functional capability, social situation and care need among elderly, **General**	Prospective & retrospective 60					x	x	x	q	x					x	x					
S17 Infants of Western Sweden	2003–	Western Sweden	8176	risk factors for sudden infant death syndrome, asthma, allergic diseases, **Specific**	Prospective Postnatal	x	x						q		x		x	x							
S18 Assessment of Lifestyle and Allergic Disease During Infancy (ALADDIN)	2004–2016	Järna and Stockholm	552	effects of environmental and lifestyle factors during pregnancy on allergies, **Specific**	Prospective Pregnancy	x	x				x	x	qi			x	x	x					x		
S19 Halland Health and Growth Study (H^2^GS)	2007–2013	Halland	2666	child health and growth from parental perspective and needs, **Specific**	Prospective Postnatal	x	x					x	q				x	x							
S20 The Swedish Environmental Longitudinal, Mother and child, Asthma and allergy (SELMA) study	2007–2015	Värmland county	2582	asthma and allergy, endocrine disrupting chemicals, early life exposures, **Specific**	Prospective Pregnancy	x	x				x		q			x	x			x		x	x		
S21 Biology, Affect, Stress, Imaging and Cognition (BASIC) cohort	2009–	Uppsala	5492	bio-psychological aetiological processes of perinatal depression (PND), **Specific**	Prospective Pregnancy	x	x				x	x	q	x	x		x			x		x	x		a
S22 The Uppsala-Stockholm Assisted Reproductive Techniques Study (UppStART)	2011–	Greater Uppsala and Stockholm municipalities	514^9^	epigenetic alterations in ART conceived children; lifestyle factors in connection to pregnancy outcomes, **Specific**	Prospective Pregnancy	x					x		q		x		x	x				x		x	
S23 Nutritional impact on Immunological maturation during Childhood in relation to the Environment (NICE)	2014–	Luleå Arctic	655	immunological maturation and allergy development, environmental exposures, **Specific**	Prospective Pregnancy	x	x				x	x	q				x	x				x			
S24 NothPop	2016–	Västerbotten	10 000	lifestyle-related diseases and behaviours, **General**	Prospective Pregnancy	x	x	x			x		q		x		x	x				x			a
**ICELAND**						1	2	3	4	5	1	2	3	4	5	6	1	2	3	4	5	1	2	3	4
I1 LIFECOURSE study	2016–2020	Whole country	2378	influence of stress on harmful behavioural outcomes among adolescents, **Specific**	Retrospective & prospective 12/13			x			x		q		x				x						

^1^We have listed main references (e.g. article, cohort webpage) of each cohort to a Appendix 3.

^2^Column “Respondents on questionnaires/interviews” refers to participants who responded to the questionnaires/surveys in order to illustrate who the active respondents were in contrast to, for instance, routinely collected anthropometric measures, such as height and weight.

^3^Uppstart participants were adult couples.

## Appendix 3: Main references of Appendix 2,The characteristics of the included birth cohorts

**DENMARK**

D1: Copenhagen Periantal Birth Cohort 1959-1967 [Internet]. Denmark: Riksargivet; 2022 [cited 2022 Apr 20]. Available from: http://dda.dk/catalogue-series/60085?lang=en

Merrick J, Teasdale TW, Merrick Y. School health screening of a birth cohort: a prospective longitudinal study. Int J Rehabil Res. 1983 Dec;6(4):461-8. doi: 10.1097/00004356-198312000-00005. PMID: 6674223.

D2: Osler M, Linneberg A, Glümer C, Jørgensen T. The cohorts at the Research Centre for Prevention and Health, formerly ‘The Glostrup Population Studies’. Int J Epidemiol. 2011 Jun;40(3):602-10. doi: 10.1093/ije/dyq041. Epub 2010 Mar 24. PMID: 20338891.

D3: The Metropolit Danish birth cohort [Internet]. The EU Joint Programme – Neurodegenerative Disease Research (JPND); 2022 [cited 2022 Apr 20]. Available from: https://www.neurodegenerationresearch.eu/cohort/the-metropolit-1953-danish-male-birth-cohort/

Osler M, Lund R, Kriegbaum M, Christensen U, Andersen AM. Cohort profile: the Metropolit 1953 Danish male birth cohort. Int J Epidemiol. 2006 Jun;35(3):541-5. doi: 10.1093/ije/dyi300. Epub 2005 Dec 23. PMID: 16377658.

D4: Hansen LG, Halken S, Høst A, Møller K, Osterballe O. Prediction of allergy from family history and cord blood IgE levels. A follow-up at the age of 5 years. Cord blood IgE. IV. Pediatr Allergy Immunol. 1993 Feb;4(1):34-40. doi: 10.1111/j.1399-3038.1993.tb00063.x. PMID: 8348254.

D5: Olsen J, Frische G, Poulsen AO, Kirchheiner H. Changing smoking, drinking, and eating behaviour among pregnant women in Denmark. Evaluation of a health campaign in a local region. Scand J Soc Med. 1989;17(4):277-80. doi: 10.1177/140349488901700404. PMID: 2602919. https://open.rsyd.dk/OpenProjects/openProject.jsp?openNo=685&lang=en

D6: Nissen SP, Kjaer HF, Høst A, Nielsen J, Halken S. The natural course of sensitization and allergic diseases from childhood to adulthood. Pediatr Allergy Immunol. 2013 Sep;24(6):549-55. doi: 10.1111/pai.12108. Epub 2013 Jul 31. PMID: 23902477.

D7: The CHEF project [Internet]. Denmark; 2022 [cited 2022 Apr 20]. Available from: http://www.chef-project.dk/

D8: Petersen SB, Olsen SF, Mølgaard C, Granström C, Cohen A, Vestergaard P, Strøm M. Maternal vitamin D status and offspring bone fractures: prospective study over two decades in Aarhus City, Denmark. PLoS One. 2014 Dec 4;9(12):e114334. doi: 10.1371/journal.pone.0114334. PMID: 25474409; PMCID: PMC4256222.

D9: Hedegaard M, Henriksen TB, Sabroe S, Secher NJ. Psychological distress in pregnancy and preterm delivery. BMJ. 1993 Jul 24;307(6898):234-9. doi: 10.1136/bmj.307.6898.234. PMID: 8369684; PMCID: PMC1678180.

ABC Biobank: Mortensen LM, Bech BH, Nohr EA, Kruhøffer M, Kjærgaard S, Uldbjerg N, Olsen J, Henriksen TB. Data resource profile: the Aarhus birth cohort biobank (ABC biobank). Int J Epidemiol. 2013 Dec;42(6):1697-701. doi: 10.1093/ije/dyt199. PMID: 24415608.

D10: Rasmussen SH, Andersen-Ranberg K, Thinggaard M, Jeune B, Skytthe A, Christiansen L, Vaupel JW, McGue M, Christensen K. Cohort Profile: The 1895, 1905, 1910 and 1915 Danish Birth Cohort Studies - secular trends in the health and functioning of the very old. Int J Epidemiol. 2017 Dec 1;46(6):1746-1746j. doi: 10.1093/ije/dyx053. PMID: 28449061; PMCID: PMC5837237.

D11: Mother-Child cohort [Internet]. Copenhagen, Denmark; 2022 [cited 2022 Apr 20]. Available from: https://edmarc.net/mother-child-cohort.html

Henriksen LS, Mathiesen BK, Assens M, Krause M, Skakkebæk NE, Juul A, Andersson AM, Hart RJ, Newnham JP, Keelan JA, Pennell C, Main KM, Frederiksen H. Use of stored serum in the study of time trends and geographical differences in exposure of pregnant women to phthalates. Environ Res. 2020 May;184:109231. doi: 10.1016/j.envres.2020.109231. Epub 2020 Feb 7. PMID: 32087443.

D12: Danish National Birth Cohort [Internet]. Copenhagen, Denmark; 2022 [cited 2022 Apr 20]. Available from:/https://www.dnbc.dk

D13: Ottosen MH. Research on the Danish Longitudinal Survey of Children (DALSC) at the Danish National Centre for Social Research. Scand J Public Health. 2011 Jul;39(7 Suppl):121-5. doi: 10.1177/1403494811399165. PMID: 21775369.

D14. Copenhagen prospective studies on asthma in childhood [Internet]. Copenhagen University Hospital; 2022 [cited 2022 Apr 20]. Available from: http://copsac.com/home/copsac-cohorts/copsac2000cohort/

D15: Eller E, Kjaer HF, Høst A, Andersen KE, Bindslev-Jensen C. Development of atopic dermatitis in the DARC birth cohort. Pediatr Allergy Immunol. 2010 Mar;21(2 Pt 1):307-14. doi: 10.1111/j.1399-3038.2009.00914.x. Epub 2009 Sep 24. PMID: 19788539.

D16: Bjerregaard P, Holm AL, Olesen I, Schnor O, Niclasen B. Ivaaq. The Greenland Inuit child cohort - a preliminary report. [Internet]. Centre for Health Research in Greenland National Institute of Health, University of Southern Denmark, and Directorate of Health, Nuuk; 2007 [cited 2022 Jan 9]. Available from: https://sdunet.dk/-/media/images/sif/udgivelser/2005/groenland/ivaag_-_the_greenland_inuit_chaild_cohort_-_a_preliminary_report.pdf

D17: Olsen EM, Rask CU, Elberling H, Jeppesen P, Clemmensen L, Munkholm A, Li XQ, Hansen MH, Rimvall MK, Linneberg A, Munch IC, Larsen M, Jørgensen T, Skovgaard AM. Cohort Profile: The Copenhagen Child Cohort Study (CCC2000). Int J Epidemiol. 2020 Apr 1;49(2):370-371 l. doi: 10.1093/ije/dyz256. PMID: 31876909.

D18: Jensen JD, Andersen JH, Winding TN. Psychological resources in adolescence and the association with labour market participation in early adulthood: a prospective cohort study. BMC Public Health. 2020 Mar 24;20(1):386. doi: 10.1186/s12889-020-08531-w. PMID: 32209059; PMCID: PMC7092559.

Winding, TN. The transition from school to work life Educational attainment and work environment in a Danish youth cohort [dissertation]. Danish Ramazzini Centre, Department of Occupational Medicine Regional Hospital, West Jutland University, Aarhus University; 2014.

D19: Copenhagen prospective studies on asthma in childhood [Internet]. Copenhagen University Hospital; 2022 [cited 2022 Apr 20]. Available from: http://copsac.com/home/copsac-cohorts/copsac2000cohort/

Bisgaard H, Vissing NH, Carson CG, Bischoff AL, Følsgaard NV, Kreiner-Møller E, Chawes BL, Stokholm J, Pedersen L, Bjarnadóttir E, Thysen AH, Nilsson E, Mortensen LJ, Olsen SF, Schjørring S, Krogfelt KA, Lauritzen L, Brix S, Bønnelykke K. Deep phenotyping of the unselected COPSAC2010 birth cohort study. Clin Exp Allergy. 2013 Dec;43(12):1384-94. doi: 10.1111/cea.12213. PMID: 24118234; PMCID: PMC4158856.

D20: Kyhl HB, Jensen TK, Barington T, Buhl S, Norberg LA, Jørgensen JS, Jensen DF, Christesen HT, Lamont RF, Husby S. The Odense Child Cohort: aims, design, and cohort profile. *Paediatr Perinat Epidemiol*. 2015 May;29(3):250-8. doi: 10.1111/ppe.12183.

D21: Wielsøe, M., Berthelsen, D., Mulvad, G., Isidor, S., Long, M., & Bonefeld-Jørgensen, E. C. (2021). Dietary habits among men and women in West Greenland: follow-up on the ACCEPT birth cohort. BMC Public Health, 21(1), 1-17. https://doi.org/10.1186/s12889-021-11359-7

D22: Gerner T, Halling A, Rasmussen Rinnov M, et al‘Barrier dysfunction in Atopic newBorns studY’ (BABY): protocol of a Danish prospective birth cohort studyBMJ Open 2020;10:e033801. doi: 10.1136/bmjopen-2019-033801

**FINLAND**

F1: Northern Finland Birth Cohorts and Arctic Biobank [Internet]. Oulu: University of Oulu, Finland; 2022 [cited 2022 Apr 20]. Available from: https://www.oulu.fi/en/university/faculties-and-units/faculty-medicine/northern-finland-birth-cohorts-and-arctic-biobank

Nordström T, Miettunen J, Auvinen J, Ala-Mursula L, Keinänen-Kiukaanniemi S, Veijola J et al. Cohort Profile: 46 years of follow-up of the Northern Finland Birth Cohort 1966 (NFBC1966). Int J Epidemiol. 2021;50(6):1786-1787j.

F2: Michelsson K, Ylinen A, Saarnivaara A, Donner M. Occurrence of risk factors in newborn infants. A study of 22359 consecutive cases. Ann Clin Res. 1978 Dec;10(6):334-6. PMID: 742834.

F3: Brannigan R, Tanskanen A, Huttunen MO, Cannon M, Leacy FP, Clarke MC. The role of prenatal stress as a pathway to personality disorder: longitudinal birth cohort study. Br J Psychiatry. 2020 Feb;216(2):85-89. doi: 10.1192/bjp.2019.190. Erratum in: Br J Psychiatry. 2020 May;216(5):285. PMID: 31488224.

F4: Raitakari OT, Juonala M, Rönnemaa T, Keltikangas-Järvinen L, Räsänen L, Pietikäinen M, Hutri-Kähönen N, Taittonen L, Jokinen E, Marniemi J, Jula A, Telama R, Kähönen M, Lehtimäki T, Akerblom HK, Viikari JS. Cohort profile: the cardiovascular risk in Young Finns Study. Int J Epidemiol. 2008 Dec;37(6):1220-6. doi: 10.1093/ije/dym225. Epub 2008 Feb 8. PMID: 18263651. DOI: 10.1093/ije/dym225

https://youngfinnsstudy.utu.fi/

F5: Fuentes RM, Notkola IL, Shemeikka S, Tuomilehto J, Nissinen A. Tracking of systolic blood pressure during childhood: a 15-year follow-up population-based family study in eastern Finland. J Hypertens. 2002 Feb;20(2):195-202. doi: 10.1097/00004872-200202000-00008. PMID: 11821703. DOI: 10.1097/00004872-200202000-00008

F6: Arvo Ylppö Longitudinal Study (AYLS) cohort from Finland. [Internet]. The European Foundation for the Care of Newborn Infants (EFCNI); 2022. [cited 2022 Apr 20]. Available from: https://www.efcni.org/news/arvo-ylppo-longitudinal-study-ayls-cohort-from-finland/

Kumpulainen SM, Heinonen K, Salonen MK, Andersson S, Wolke D, Kajantie E, Eriksson JG, Raikkonen K. Childhood cognitive ability and body composition in adulthood. Nutr Diabetes. 2016 Aug 15;6(8):e223. doi: 10.1038/nutd.2016.30. PMID: 27525818; PMCID: PMC5022144.

F7: Northern Finland Birth Cohorts and Arctic Biobank [Internet]. Oulu: University of Oulu, Finland; 2022 [cited 2022 Apr 20]. Available from: https://www.oulu.fi/en/university/faculties-and-units/faculty-medicine/northern-finland-birth-cohorts-and-arctic-biobank

F8: Gyllenberg D, Sourander A, Niemelä S, Helenius H, Sillanmäki L, Piha J, Kumpulainen K, Tamminen T, Moilanen I, Almqvist F. Childhood predictors of later psychiatric hospital treatment: findings from the Finnish 1981 birth cohort study. Eur Child Adolesc Psychiatry. 2010 Nov;19(11):823-33. doi: 10.1007/s00787-010-0129-1. Epub 2010 Sep 7. PMID: 20821264.

F9: The Diabetes Prediction and Prevention Nutrition Study (DIPP) [Internet]. University of Tampere, Finland; 2022. [cited 2022 Apr 20]. Available from:https://www.tuni.fi/en/research/diabetes-prediction-and-prevention-nutrition-study-dipp

F10: Helsinki Birth Cohort Study (HBCS) - Idefix [Internet]. Finnish Institute of Health and Welfare; 2022. [cited 2022 Apr 20]. Available from:https://thl.fi/en/web/thlfi-en/research-and-development/research-and-projects/helsinki-birth-cohort-study-hbcs-idefix

Eriksson JG. Early growth, and coronary heart disease and type 2 diabetes: experiences from the Helsinki Birth Cohort Studies. Int J Obes (Lond). 2006 Dec;30 Suppl 4:S18-22. doi: 10.1038/sj.ijo.0803515. Erratum in: Int J Obes (Lond). 2010 Jul;34(7):1230. PMID: 17133231.

F11: Strandberg TE, Järvenpää AL, Vanhanen H, McKeigue PM. Birth outcome in relation to licorice consumption during pregnancy. Am J Epidemiol. 2001 Jun 1;153(11):1085-8. doi: 10.1093/aje/153.11.1085. PMID: 11390327.

F12: PIPARI Pienipainoiset riskilapset [Internet]. University of Turku, Finland; 2022. [cited 2022 Apr 20]. Available from: https://sites.utu.fi/pipari/en/

F13: Eero Kajantie, Petteri Hovi, Katri Räikkönen, Anu-Katriina Pesonen, Kati Heinonen, Anna-Liisa Järvenpää, Johan G Eriksson, Sonja Strang-Karlsson, Sture Andersson; Young Adults With Very Low Birth Weight: Leaving the Parental Home and Sexual Relationships—Helsinki Study of Very Low Birth Weight Adults. Pediatrics July 2008; 122 (1): e62–e72. 10.1542/peds.2007-3858 https://doi.org/10.1542/peds.2007-3858

https://thl.fi/en/web/thlfi-en/research-and-development/research-and-projects/helsinki-study-of-very-low-birth-weight-adults-hesva-

F14: Lagström H, Rautava P, Kaljonen A, Räihä H, Pihlaja P, Korpilahti P, Peltola V, Rautakoski P, Österbacka E, Simell O, Niemi P. Cohort profile: Steps to the healthy development and well-being of children (the STEPS Study). Int J Epidemiol. 2013 Oct;42(5):1273-84. doi: 10.1093/ije/dys150. Epub 2012 Nov 9. PMID: 23143610.

F15: ESTER - Preterm Birth and Early Life Programming of Adult Health and Disease [Internet]. ReCap Preterm; 2022. [cited 2022 Apr 20]. Available from: https://platform.recap-preterm.eu/pub/study/ester

Sipola-Leppänen M, Vääräsmäki M, Tikanmäki M, Matinolli HM, Miettola S, Hovi P, Wehkalampi K, Ruokonen A, Sundvall J, Pouta A, Eriksson JG, Järvelin MR, Kajantie E. Cardiometabolic risk factors in young adults who were born preterm. Am J Epidemiol. 2015 Jun 1;181(11):861-73. doi: 10.1093/aje/kwu443. Epub 2015 May 5. PMID: 25947956; PMCID: PMC4445394.

FI16: Paavonen, E. J., Saarenpää-Heikkilä, O., Pölkki, P., Kylliäinen, A., Porkka-Heiskanen, T., & Paunio, T. (2017). Maternal and paternal sleep during pregnancy in the Child-sleep birth cohort. Sleep medicine, 29, 47-56.

F17: FinnBrain Research [Internet]. University of Turku, Finland; 2022. [cited 2022 Apr 20]. Available from: https://sites.utu.fi/finnbrain/en/

Karlsson L, Tolvanen M, Scheinin NM, Uusitupa HM, Korja R, Ekholm E, Tuulari JJ, Pajulo M, Huotilainen M, Paunio T, Karlsson H; FinnBrain Birth Cohort Study Group. Cohort Profile: The FinnBrain Birth Cohort Study (FinnBrain). Int J Epidemiol. 2018 Feb 1;47(1):15-16j. doi: 10.1093/ije/dyx173. PMID: 29025073.

F18: KuBiCo - Kuopio Birth Cohort [Internet]. University of Eastern Finland, Finland; 2022. [cited 2022 Apr 20]. Available from: http://www.kubico.fi/en_presentation.aspx

F19: Kielenniva, K, Ainonen, S, Vänni, P, et al. Microbiota of the first-pass meconium and subsequent atopic and allergic disorders in children. Clin Exp Allergy. 2022; 52: 684– 696. doi:10.1111/cea.14117 https://doi.org/10.1111/cea.14117

F20: Korpela K, Dikareva E, Hanski E, Kolho KL, de Vos WM, Salonen A. Cohort profile: Finnish Health and Early Life Microbiota (HELMi) longitudinal birth cohort. BMJ Open. 2019 Jun 27;9(6):e028500. doi: 10.1136/bmjopen-2018-028500. PMID: 31253623; PMCID: PMC6609051.

**NORWAY**

N1: Kinge FO. Work and disability at the age of 30 years. A sociomedical study of a birth-cohort from Bergen. III. Disability as related to medical, psychological and educational background. Scand J Soc Med. 1978;6(3):131-6. doi: 10.1177/140349487800600306. PMID: 152972.

N2: Holst D, Schuller AA. Equality in adults’ oral health in Norway. Cohort and cross-sectional results over 33 years. Community Dent Oral Epidemiol. 2011 Dec;39(6):488-97. doi: 10.1111/j.1600-0528.2011.00624.x. Epub 2011 Jul 14. PMID: 21756267.

N3: Rossow I, Kjaernes U, Holst D. Patterns of sugar consumption in early childhood. Community Dent Oral Epidemiol. 1990 Feb;18(1):12-6. doi: 10.1111/j.1600-0528.1990.tb00654.x. PMID: 2297974.

N4: Lødrup Carlsen KC. The environment and childhood asthma (ECA) study in Oslo: ECA-1 and ECA-2. Pediatr Allergy Immunol. 2002;13(s15):29-31. doi: 10.1034/j.1399-3038.13.s.15.2.x. PMID: 12688621.

N5: Norwegian Mother, Father and Child Cohort Study (MoBa) [Internet]. Norwegian institute of public health; 2022. [cited 2022 Apr 20]. Available from: https://www.fhi.no/en/studies/moba/

Magnus P, Birke C, Vejrup K, Haugan A, Alsaker E, Daltveit AK, Handal M, Haugen M, Høiseth G, Knudsen GP, Paltiel L, Schreuder P, Tambs K, Vold L, Stoltenberg C. Cohort Profile Update: The Norwegian Mother and Child Cohort Study (MoBa). Int J Epidemiol. 2016 Apr;45(2):382-8. doi: 10.1093/ije/dyw029. Epub 2016 Apr 10. PMID: 27063603.

N6: MIDIA (Environmental causes of type 1 diabetes) - project description [Internet]. Norwegian institute of public health; 2022. [cited 2022 Apr 20]. Available from: https://www.fhi.no/en/cristin-projects/ongoing/midia-environmental-causes-of-type-1-diabetes/

Jørgenrud B, Stene LC, Tapia G, Bøås H, Pepaj M, Berg JP, Thorsby PM, Orešič M, Hyötyläinen T, Rønningen KS. Longitudinal plasma metabolic profiles, infant feeding, and islet autoimmunity in the MIDIA study. Pediatr Diabetes. 2017 Mar;18(2):111-119. doi: 10.1111/pedi.12360. Epub 2016 Jan 21. PMID: 26791677.

N7: The Norwegian Human Milk Study - HUMIS - project description [Internet]. Norwegian institute of public health; 2022. [cited 2022 Apr 20]. Available from:https://www.fhi.no/en/projects/HUMIS/

Vollset M, Iszatt N, Enger Ø, Gjengedal ELF, Eggesbø M. Concentration of mercury, cadmium, and lead in breast milk from Norwegian mothers: Association with dietary habits, amalgam and other factors. Sci Total Environ. 2019 Aug 10;677:466-473. doi: 10.1016/j.scitotenv.2019.04.252. Epub 2019 Apr 19. PMID: 31063889.

N8: Norwegian Microbiota Study (NoMIC) [Internet]. Norwegian institute of public health; 2022. [cited 2022 Apr 20]. Available from: https://www.fhi.no/en/studies/nomic/

Eggesbø M, Moen B, Peddada S, Baird D, Rugtveit J, Midtvedt T, Bushel PR, Sekelja M, Rudi K. Development of gut microbiota in infants not exposed to medical interventions. APMIS. 2011 Jan;119(1):17-35. doi: 10.1111/j.1600-0463.2010.02688.x. Epub 2010 Oct 25. PMID: 21143523; PMCID: PMC3058492.

N9: Steinsbekk S, Wichstrøm L. Cohort Profile: The Trondheim Early Secure Study (TESS)-a study of mental health, psychosocial development and health behaviour from preschool to adolescence. Int J Epidemiol. 2018 Oct 1;47(5):1401-1401i. doi: 10.1093/ije/dyy190. PMID: 30215720.

N10: The Ahus Birth Cohort [Internet]. Faculty of Medicine; Institute of Clinical Medicine; University of Oslo; Norway; 2022. [cited 2022 Apr 20]. Available from: https://www.med.uio.no/klinmed/english/research/projects/ahus-birth-cohort/

Deforges C, Stuijfzand S, Noël Y, Robertson M, Breines Simonsen T, Eberhard-Gran M, Garthus-Niegel S, Horsch A. The relationship between early administration of morphine or nitrous oxide gas and PTSD symptom development. J Affect Disord. 2021 Feb 15;281:557-566. doi: 10.1016/j.jad.2020.12.051. Epub 2020 Dec 16. PMID: 33421836.

N11: NorFlu [Internet]. Norwegian institute of public health; 2022. [cited 2022 Apr 20]. Available from: https://www.fhi.no/en/studies/norflu/

Borren I, Tambs K, Gustavson K, Schjølberg S, Eriksen W, Håberg SE, Hungnes O, Mjaaland S, Trogstad LIS. Early prenatal exposure to pandemic influenza A (H1N1) infection and child psychomotor development at 6 months - A population-based cohort study. Early Hum Dev. 2018 Jul;122:1-7. doi: 10.1016/j.earlhumdev.2018.05.005. Epub 2018 May 24. PMID: 29803166.

N12: Trygve Berge, Thea Vigen, Mohammad Osman Pervez, Haakon Ihle-Hansen, Magnus Nakrem Lyngbakken, Torbjørn Omland, Pål Smith, Kjetil Steine, Helge Røsjø, Arnljot Tveit & on behalf of the ACE 1950 Study Group (2015) Heart and Brain Interactions – the Akershus Cardiac Examination (ACE) 1950 Study Design, Scandinavian Cardiovascular Journal, 49:6, 308-315, DOI: 10.3109/14017431.2015.1086813

https://ace1950.no/prosjektorganisasjon/

**SWEDEN**

S1: Fu M, Rosengren A, Thunström E, Mandalenakis Z, Welin L, Caidahl K, Pivodic A, Zhong Y, Ergatoudes C, Morales D, Welin C, Svärdsudd K, Dellborg M, Hansson PO. Although Coronary Mortality Has Decreased, Rates of Cardiovascular Disease Remain High: 21 Years of Follow-Up Comparing Cohorts of Men Born in 1913 With Men Born in 1943. J Am Heart Assoc. 2018 Apr 19;7(9):e008769. doi: 10.1161/JAHA.118.008769. PMID: 29674335; PMCID: PMC6015276.

S2: Sten-Åke Stenberg, Denny Vågerö, Cohort Profile: The Stockholm birth cohort of 1953, International Journal of Epidemiology, Volume 35, Issue 3, June 2006, Pages 546–548, https://doi.org/10.1093/ije/dyi310; https://www.stockholmbirthcohort.su.se/about-the-project/original-data-1953-1983

S3: Nilsson PM, Hofvendahl S, Hofvendahl E, Brandt L, Ekbom A. Smoking in pregnancy in relation to gender and adult mortality risk in offspring: the Helsingborg Birth Cohort Study. Scand J Public Health. 2006;34(6):660-4. doi: 10.1080/14034940600607509. PMID: 17132600.

S4: Gudmundsson P, Lindwall M, Gustafson DR, Östling S, Hällström T, Waern M, Skoog I. Longitudinal associations between physical activity and depression scores in Swedish women followed 32 years. Acta Psychiatr Scand. 2015 Dec;132(6):451-8. doi: 10.1111/acps.12419. Epub 2015 Apr 11. PMID: 25865488; PMCID: PMC4600636.

S5: Mellbin T, Sundelin C, Vuille JC. Growing up in Uppsala. The role of public services in identification and treatment of health and adjustment problems. Part I. Definition and classification of dependent variables. Acta Paediatr. 1992 May;81(5):417-23. doi: 10.1111/j.1651-2227.1992.tb12261.x. PMID: 1498509.

S6: Rydberg Sterner T, Ahlner F, Blennow K, Dahlin-Ivanoff S, Falk H, Havstam Johansson L, et al. The Gothenburg H70 Birth cohort study 2014-16: design, methods and study population. Eur J Epidemiol. 2019 Feb;34(2):191-209. doi: 10.1007/s10654-018-0459-8. Epub 2018 Nov 13. PMID: 30421322; PMCID: PMC6373310.

S7: The Gothenburg H70 Birth cohort study [Internet]. University of Gothenburg, Sweden; 2022. [cited 2022 Apr 20]. Available from: https://www.gu.se/en/research/the-gothenburg-h70-birth-cohort-study

S8: Vogt H, Bråbäck L, Zetterström O, Zara K, Fälth-Magnusson K, Nilsson L. Asthma Heredity, Cord Blood IgE and Asthma-Related Symptoms and Medication in Adulthood: A Long-Term Follow-Up in a Swedish Birth Cohort. PLoS One. 2013 Jun 21;8(6):e66777. doi: 10.1371/journal.pone.0066777. PMID: 23805276; PMCID: PMC3689672.

S9: Henriksson KM, Lindblad U, Gullberg B, Agren B, Nilsson-Ehle P, Råstam L. Body composition, ethnicity and alcohol consumption as determinants for the development of blood pressure in a birth cohort of young middle-aged men. Eur J Epidemiol. 2003;18(10):955-63. doi: 10.1023/a:1025851006527. PMID: 14598926.

S10: BAMSE Project [Internet]. Institute of Environmental medicine, karolinska Institutet, Sweden; 2022. [cited 2022 Apr 20]. Available from: https://ki.se/en/imm/bamse-project

S11: Agnafors S, Sydsjö G, Dekeyser L, Svedin CG. Symptoms of depression postpartum and 12 years later-associations to child mental health at 12 years of age. Matern Child Health J. 2013 Apr;17(3):405-14. doi: 10.1007/s10995-012-0985-z. PMID: 22466717.

S12: Al-Tamprouri C, Malin B, Bill H, Lennart B, Anna S. Cat and dog ownership during/after the first year of life and risk for sensitization and reported allergy symptoms at age 13. Immun Inflamm Dis. 2019 Dec;7(4):250-257. doi: 10.1002/iid3.267. Epub 2019 Aug 29. PMID: 31464382; PMCID: PMC6842813.

S13: Klintberg, B., Berglund, N., Lilja, G., Wickman, M., & van Hage-Hamsten, M. (2001). Fewer allergic respiratory disorders among farmers’ children in a closed birth cohort from Sweden. European Respiratory Journal, 17(6), 1151-1157.

S14: ABIS All barn I sydostra Sverige [Internet]. Institutionen för klinisk och experimentell medicin (IKE), Sweden; 2022. [cited 2022 Apr 20]. Available from: https://www.abis-studien.se/hem/english-11100423

S15: Diabetes Prediction in Skåne- DiPiS [Internet]. Swedish national data service; 2022. [cited 2022 Apr 20]. Available from: https://snd.gu.se/en/catalogue/study/ext0081#description

S16: Good Aging in Skåne database - part of National E-Infrastructure for Ageing Research (NEAR) [Internet]. Lund University, Sweden; 2022. [cited 2022 Apr 20]. Available from: https://portal.research.lu.se/en/equipments/good-aging-in-sk%C3%A5ne-database

S17: Goksör E, Alm B, Thengilsdottir H, Pettersson R, Åberg N, Wennergren G. Preschool wheeze - impact of early fish introduction and neonatal antibiotics. Acta Paediatr. 2011 Dec;100(12):1561-6. doi: 10.1111/j.1651-2227.2011.02411.x. Epub 2011 Jul 30. PMID: 21767307.

S18: Stenius F, Swartz J, Lilja G, Borres M, Bottai M, Pershagen G, Scheynius A, Alm J. Lifestyle factors and sensitization in children - the ALADDIN birth cohort. Allergy. 2011 Oct;66(10):1330-8. doi: 10.1111/j.1398-9995.2011.02662.x. Epub 2011 Jun 9. PMID: 21651566.

S19: Roswall J, Almqvist-Tangen G, Holmén A, Alm B, Bergman S, Dahlgren J, Strömberg U. Overweight at four years of age in a Swedish birth cohort: influence of neighbourhood-level purchasing power. BMC Public Health. 2016 Jul 11;16:546. doi: 10.1186/s12889-016-3252-1. PMID: 27400741; PMCID: PMC4940903.

S20: Bornehag CG, Moniruzzaman S, Larsson M, Lindström CB, Hasselgren M, Bodin A, von Kobyletzkic LB, Carlstedt F, Lundin F, Nånberg E, Jönsson BA, Sigsgaard T, Janson S. The SELMA study: a birth cohort study in Sweden following more than 2000 mother-child pairs. Paediatr Perinat Epidemiol. 2012 Sep;26(5):456-67. doi: 10.1111/j.1365-3016.2012.01314.x. Epub 2012 Jul 23. PMID: 22882790.

S21: Basic Studien – biology, affect, stress, imaging and cognition [Internet]. 2022. [cited 2022 Apr 20]. Available from: https://www.basicstudie.se/copy-of-om-basic-studie

Axfors C, Bränn E, Henriksson HE, Hellgren C, Kunovac Kallak T, Fransson E, Lager S, Iliadis SI, Sylvén S, Papadopoulos FC, Ekselius L, Sundström-Poromaa I, Skalkidou A. Cohort profile: the Biology, Affect, Stress, Imaging and Cognition (BASIC) study on perinatal depression in a population-based Swedish cohort. BMJ Open. 2019 Oct 22;9(10):e031514. doi: 10.1136/bmjopen-2019-031514. PMID: 31641004; PMCID: PMC6830667.

S22: Iliadou AN, Öberg AS, Pege J, Rodriguez-Wallberg KA, Olofsson JI, Holte J, Wramsby H, Wramsby M, Cnattingius S, Cesta CE. The Uppsala-Stockholm Assisted Reproductive Techniques (UppStART) study. BMJ Open. 2019 Aug 28;9(8):e028866. doi: 10.1136/bmjopen-2018-028866. PMID: 31467051; PMCID: PMC6720339.

S23: Barman M, Murray F, Bernardi AI, Broberg K, Bölte S, Hesselmar B, Jacobsson B, Jonsson K, Kippler M, Rabe H, Ross AB, Sjöberg F, Strömberg N, Vahter M, Wold AE, Sandberg AS, Sandin A. Nutritional impact on Immunological maturation during Childhood in relation to the Environment (NICE): a prospective birth cohort in northern Sweden. BMJ Open. 2018 Oct 21;8(10):e022013. doi: 10.1136/bmjopen-2018-022013. PMID: 30344169; PMCID: PMC6196815.

S24: Lundkvist E, Stoltz Sjöström E, Lundberg R, Silfverdal S-A, West CE, Domellöf M. Fruit Pouch Consumption and Dietary Patterns Related to BMIz at 18 Months of Age. Nutrients. 2021; 13(7):2265. https://doi.org/10.3390/nu13072265

https://www.umu.se/en/research/infrastructure/northpop/

**ICELAND**

I1: Halldorsdottir T, Kristjansson AL, Asgeirsdottir BB, Thorisdottir IE, Sigfusson J, Tolgyes EMJ, Valdimarsdottir HB, Allegrante J, Sigfusdottir ID. A multi-level developmental approach towards understanding adolescent mental health and behaviour: rationale, design and methods of the LIFECOURSE study in Iceland. Soc Psychiatry Psychiatr Epidemiol. 2021 Mar;56(3):519-529. doi: 10.1007/s00127-020-01995-6. Epub 2020 Nov 25. PMID: 33236265.

## Appendix 4 Prisma 2020 for Abstract checklist

**Table:**

Section and Topic	Item #	Checklist item	Reported (Yes/No)
**TITLE**			
Title	1	Identify the report as a systematic review.	Yes
**BACKGROUND**			
Objectives	2	Provide an explicit statement of the main objective(s) or question(s) the review addresses.	Yes
**METHODS**			
Eligibility criteria	3	Specify the inclusion and exclusion criteria for the review.	No
Information sources	4	Specify the information sources (e.g. databases, registers) used to identify studies and the date when each was last searched.	No
Risk of bias	5	Specify the methods used to assess risk of bias in the included studies.	NA
Synthesis of results	6	Specify the methods used to present and synthesise results.	No
**RESULTS**			
Included studies	7	Give the total number of included studies and participants and summarise relevant characteristics of studies.	Yes
Synthesis of results	8	Present results for main outcomes, preferably indicating the number of included studies and participants for each. If meta-analysis was done, report the summary estimate and confidence/credible interval. If comparing groups, indicate the direction of the effect (i.e. which group is favoured).	Yes
**DISCUSSION**			
Limitations of evidence	9	Provide a brief summary of the limitations of the evidence included in the review (e.g. study risk of bias, inconsistency and imprecision).	No
Interpretation	10	Provide a general interpretation of the results and important implications.	Yes
**OTHER**			
Funding	11	Specify the primary source of funding for the review.	No (in abstract)
Registration	12	Provide the register name and registration number.	NA

From: Page MJ, McKenzie JE, Bossuyt PM, Boutron I, Hoffmann TC, Mulrow CD, et al. The PRISMA 2020 statement: an updated guideline for reporting systematic reviews. BMJ 2021;372:n71. doi: 10.1136/bmj.n71 For more information, visit: http://www.prisma-statement.org/

## Appendix 5 Prisma 2020 checklist

**Table:**

Section and Topic	Item #	Checklist item	Location where item is reported
**TITLE**			
Title	1	Identify the report as a systematic review.	pg 1
**ABSTRACT**			
Abstract	2	See the PRISMA 2020 for Abstracts checklist.	
**INTRODUCTION**			
Rationale	3	Describe the rationale for the review in the context of existing knowledge.	pg 2–3
Objectives	4	Provide an explicit statement of the objective(s) or question(s) the review addresses.	pg 3
**METHODS**			
Eligibility criteria	5	Specify the inclusion and exclusion criteria for the review and how studies were grouped for the syntheses.	pg 3
Information sources	6	Specify all databases, registers, websites, organisations, reference lists and other sources searched or consulted to identify studies. Specify the date when each source was last searched or consulted.	pg 3–4 and Appendix 1
Search strategy	7	Present the full search strategies for all databases, registers and websites, including any filters and limits used.	Appendix 1
Selection process	8	Specify the methods used to decide whether a study met the inclusion criteria of the review, including how many reviewers screened each record and each report retrieved, whether they worked independently, and if applicable, details of automation tools used in the process.	pg 3–4
Data collection process	9	Specify the methods used to collect data from reports, including how many reviewers collected data from each report, whether they worked independently, any processes for obtaining or confirming data from study investigators, and if applicable, details of automation tools used in the process.	pg 3–4
Data items	10a	List and define all outcomes for which data were sought. Specify whether all results that were compatible with each outcome domain in each study were sought (e.g. for all measures, time points, analyses), and if not, the methods used to decide which results to collect.	pg 4
	10b	List and define all other variables for which data were sought (e.g. participant and intervention characteristics, funding sources). Describe any assumptions made about any missing or unclear information.	pg 3–4
Study risk of bias assessment	11	Specify the methods used to assess risk of bias in the included studies, including details of the tool(s) used, how many reviewers assessed each study and whether they worked independently, and if applicable, details of automation tools used in the process.	NA
Effect measures	12	Specify for each outcome the effect measure(s) (e.g. risk ratio, mean difference) used in the synthesis or presentation of results.	NA
Synthesis methods	13a	Describe the processes used to decide which studies were eligible for each synthesis (e.g. tabulating the study intervention characteristics and comparing against the planned groups for each synthesis (item #5)).	NA
	13b	Describe any methods required to prepare the data for presentation or synthesis, such as handling of missing summary statistics, or data conversions.	NA
	13c	Describe any methods used to tabulate or visually display results of individual studies and syntheses.	NA
	13d	Describe any methods used to synthesise results and provide a rationale for the choice(s). If meta-analysis was performed, describe the model(s), method(s) to identify the presence and extent of statistical heterogeneity, and software package(s) used.	NA
	13e	Describe any methods used to explore possible causes of heterogeneity among study results (e.g. subgroup analysis, meta-regression).	NA
	13f	Describe any sensitivity analyses conducted to assess robustness of the synthesised results.	NA
Reporting bias assessment	14	Describe any methods used to assess risk of bias due to missing results in a synthesis (arising from reporting biases).	NA
Certainty assessment	15	Describe any methods used to assess certainty (or confidence) in the body of evidence for an outcome.	NA
**RESULTS**			
Study selection	16a	Describe the results of the search and selection process, from the number of records identified in the search to the number of studies included in the review, ideally using a flow diagram.	pg 4, Figure 1
	16b	Cite studies that might appear to meet the inclusion criteria, but which were excluded, and explain why they were excluded.	Not possible due to space limitations
Study characteristics	17	Cite each included study and present its characteristics.	Appendix 2
Risk of bias in studies	18	Present assessments of risk of bias for each included study.	NA
Results of individual studies	19	For all outcomes, present, for each study: (a) summary statistics for each group (where appropriate) and (b) an effect estimate and its precision (e.g. confidence/credible interval), ideally using structured tables or plots.	NA
Results of syntheses	20a	For each synthesis, briefly summarise the characteristics and risk of bias among contributing studies.	NA
	20b	Present results of all statistical syntheses conducted. If meta-analysis was done, present for each the summary estimate and its precision (e.g. confidence/credible interval) and measures of statistical heterogeneity. If comparing groups, describe the direction of the effect.	NA
	20c	Present results of all investigations of possible causes of heterogeneity among study results.	NA
	20d	Present results of all sensitivity analyses conducted to assess the robustness of the synthesised results.	NA
Reporting biases	21	Present assessments of risk of bias due to missing results (arising from reporting biases) for each synthesis assessed.	NA
Certainty of evidence	22	Present assessments of certainty (or confidence) in the body of evidence for each outcome assessed.	NA
**DISCUSSION**			
Discussion	23a	Provide a general interpretation of the results in the context of other evidence.	pg 10–13
	23b	Discuss any limitations of the evidence included in the review.	pg 12
	23c	Discuss any limitations of the review processes used.	pg 12
	23d	Discuss implications of the results for practice, policy, and future research.	pg 12–13
**OTHER INFORMATION**			
Registration and protocol	24a	Provide registration information for the review, including register name and registration number, or state that the review was not registered.	Appendix 1
	24b	Indicate where the review protocol can be accessed, or state that a protocol was not prepared.	Appendix 1
	24c	Describe and explain any amendments to information provided at registration or in the protocol.	NA
Support	25	Describe sources of financial or non-financial support for the review, and the role of the funders or sponsors in the review.	pg 13
Competing interests	26	Declare any competing interests of review authors.	pg 13
Availability of data, code and other materials	27	Report which of the following are publicly available and where they can be found: template data collection forms; data extracted from included studies; data used for all analyses; analytic code; any other materials used in the review.	Appendix 1

*From:* Page MJ, McKenzie JE, Bossuyt PM, Boutron I, Hoffmann TC, Mulrow CD, et al. The PRISMA 2020 statement: an updated guideline for reporting systematic reviews. BMJ 2021;372:n71. doi: 10.1136/bmj.n71
